# Fine-scale population structure and evidence for local adaptation in Australian giant black tiger shrimp (*Penaeus monodon*) using SNP analysis

**DOI:** 10.1186/s12864-020-07084-x

**Published:** 2020-09-29

**Authors:** Nga T. T. Vu, Kyall R. Zenger, Jarrod L. Guppy, Melony J. Sellars, Catarina N. S. Silva, Shannon R. Kjeldsen, Dean R. Jerry

**Affiliations:** 1 Australian Research Council Industrial Transformation Research Hub for Advanced Prawn Breeding, James Cook University, Townsville, QLD 4811 Australia; 2 Centre for Sustainable Tropical Fisheries and Aquaculture, College of Science and Engineering, James Cook University, Townsville, QLD 4811 Australia; 3 CSIRO Agriculture & Food, Integrated Sustainable Aquaculture Production Program, Queensland Bioscience Precinct, St Lucia, 4067 Australia; 4 Present address: Genics Pty Ltd, Level 5, Gehrmann Building. 60 Research Road, St Lucia, QLD 4067 Australia; 5 Tropical Futures Institute, James Cook University, Singapore, Singapore

**Keywords:** *Aquaculture*, Population genetics, Prawn, Functional annotation, Genotype–environment interaction

## Abstract

**Background:**

Restrictions to gene flow, genetic drift, and divergent selection associated with different environments are significant drivers of genetic differentiation. The black tiger shrimp (*Penaeus monodon*), is widely distributed throughout the Indian and Pacific Oceans including along the western, northern and eastern coastline of Australia, where it is an important aquaculture and fishery species. Understanding the genetic structure and the influence of environmental factors leading to adaptive differences among populations of this species is important for farm genetic improvement programs and sustainable fisheries management.

**Results:**

Based on 278 individuals obtained from seven geographically disparate Australian locations, 10,624 high-quality SNP loci were used to characterize genetic diversity, population structure, genetic connectivity, and adaptive divergence. Significant population structure and differentiation were revealed among wild populations (average *F*_*ST*_ = 0.001–0.107; *p* <  0.05). Eighty-nine putatively outlier SNPs were identified to be potentially associated with environmental variables by using both population differentiation (BayeScan and PCAdapt) and environmental association (redundancy analysis and latent factor mixed model) analysis methods. Clear population structure with similar spatial patterns were observed in both neutral and outlier markers with three genetically distinct groups identified (north Queensland, Northern Territory, and Western Australia). Redundancy, partial redundancy, and multiple regression on distance matrices analyses revealed that both geographical distance and environmental factors interact to generate the structure observed across Australian *P. monodon* populations.

**Conclusion:**

This study provides new insights on genetic population structure of Australian *P. monodon* in the face of environmental changes, which can be used to advance sustainable fisheries management and aquaculture breeding programs.

## Background

Local adaptation can occur when geographically discrete environmental conditions impose selection pressure on resident populations of widespread species. Within heterogeneous populations, local adaptation can arise when strong environmental selection pressure outweighs the influence of genetic drift and gene flow towards homogeneity [1]. The marine environment provides sufficient opportunities for homogenizing gene flow through passive dispersal of eggs and larvae with ocean currents and adult mobility [2]. Resident populations of discrete marine environments can initially differ genetically at a few sites in their genomes (e.g., allele frequency differences [3];, or can change rapidly to adapt to a new environment due to intense selection pressure [1, 4]. Thus, understanding how environmental features shape the genetic structure of populations is crucial because it helps to determine how populations evolve, along with the extent and scale of local adaptation [5–7]. Recently, local adaptation of marine organisms to environmental conditions has been investigated in commercially important marine invertebrates, including greenlip abalone (*Haliotis laevigata*) [8], eastern oyster (*Crassostrea virginica*) [9], American lobster (*Homarus americanus*) [10], and New Zealand scallop (*Pecten novaezelandiae*) [11]. However, to date, the influence of marine environmental conditions on the wild-type genetic diversity and structure of Australian giant black tiger shrimp (*Penaeus monodon;* Fabricius, 1798), is unknown.

Single nucleotide polymorphisms (SNP) have been demonstrated to be the most prevalent type of polymorphism within investigated genomes [4]. Moreover, SNP are becoming the marker of choice in genetic diversity, population genetics, and seascape genomics studies [12]. Additionally, genetic techniques offer a variety of methods using SNP datasets to detect putatively adaptive loci and assess how environmental parameters influence the extent of genetic variation within and among populations [13–18]. The application of these analyses has been divided into two main approaches: population differentiation (PD) and environmental association (EA). More specifically, the PD approach addresses traditional population differentiation by estimating the inherent variance in pairwise genetic differentiation values (*F*_*ST*_) of each locus across the genome without assuming the effect of environmental variables on outlier SNP generation [13, 19]. Alternatively, the EA approach addresses environmental influence on outlier SNP generation by identifying candidate adaptive loci that covary with environmental factors based on allele frequency [20, 21]. EA applications are especially promising to detect loci under putative selection, because they do not require phenotypic nor experimental data, can define the contribution of environmental variables to adaptive genetic variation, and detect weak multilocus responses to environmental conditions [20–23]. Given the unique advantages and disadvantages of each approach, unique combinations of PD and EA may help to reduce the number of false negatives and promote uncovering potential genomic footprints of selection [20]. For example, both PD and EA methods have been employed for the successful detection of putatively adaptive loci and assessment of environmental parameter influence on genetic variation within and among populations [7, 10, 20, 24].

*Penaeus monodon* is widely distributed throughout the Indo-Pacific and is found along the western, northern, and eastern coasts of Australia [25–27]. The life history of *P. monodon* comprises an offshore planktonic larval phase, estuarine juvenile and adolescent phases, and an inshore adult phase [27]. Larvae and post-larvae can display daily vertical migration behaviours in synchrony with tidal current [28, 29]. A tagging study by Gribble et al. [26] revealed that shrimp in the Trinity Inlet closure in north Queensland moved an average distance of 31 km north (5–100 km) over an average of 74 days (10–111 days). Forbes et al. [30] found evidence for high gene flow among *P. monodon* samples collected in Madagascar, Mozambique, and South Africa, despite geographic separations of up to 2000 km. Thus, *P. monodon* exhibits the capacity for moderate to high-level gene flow (i.e., genetic connectivity) across large geographic distances. In Australia, previous genetic work on *P. monodon* determined the existence of population structure using microsatellites, allozymes and mitochondrial DNA (mtDNA) markers [25, 31–33]. These studies suggested that there was no genetic differentiation between northern and eastern populations, but that *P. monodon* from Western Australia was a separate genetic stock with reduced a number of allelic due to colonisation by east coast *P. monodon* individuals. Additionally, another previous study of Indo-Pacific *P. monodon* presented that *P. monodon* from Northern Territory separated into a discrete cluster while Queensland and Western Australia grouped with Thailand, Palau, PNG, Taiwan, Philippines, and Vietnam (Bac Lieu and Can Tho) in one cluster [34].

The main objective of this study is to resolve the population genetic structure of *P. monodon* in Australia using a high-resolution genome-wide SNP approach and identify any genomic signatures of local adaptation among geographically and environmentally discrete populations. More specifically, this study aimed to (1) assess the levels of gene flow, genetic diversity, and population structure among geographically discrete populations of Australian *P. monodon* for evidence of local adaptation, and (2) predict which environmental factors are most likely to influence Australian *P. monodon* population structure (i.e., drive local adaption). The implications of *P. monodon* genetic structure and local adaptation for management and conservation of Australian populations are discussed.

## Results

### Genotyping and SNP quality control

A total of 126,511 unique SNPs were obtained for each *P. monodon* individual (*n* = 283; see Methods) by DArTseq™ sequencing (see Additional file 1). Initial quality control assessment of DArTseq™ data using *dartQC* removed 115,811 SNPs or 91.5% of loci (Table 1). The remaining 10,700 loci were then tested for LD and HWE, which removed 76 and 0 SNPs, respectively. Following genotype filtering, five individuals (two and three individuals from Gulf of Carpentaria and Nickol Bay populations, respectively) were removed due to high rates of missing data (> 40%). A final set of 10,624 SNPs for each retained *P. monodon* individual (*n* = 278) were subjected to further analyses (see Additional files 1 and 2).

**Table 1:** Filtering steps and SNPs counts retained after each step

Steps	Retained SNP count
Initial potential SNPs	126,511
Average read depth of ≥7	126,511
Replication average ≥ 0.9	122,037
Call rate of ≥80%	23,845
Similar sequence clusters of ≤0.95	19,328
Minor allele frequency (MAF) of ≥0.02	10,700
LD filters with a correlation coefficient (r^2^) threshold of 0.2	10,624
HWE filters (*p* ≥ 0.0001)	10,624
Retained SNPs for further analysis	10,624

### Population genetic diversity

For the retained 10,624 SNPs, mean allelic richness values (A_R_) ranged from 1.61 ± 0.48 for Nickol Bay (Western Australia) population to 1.75 ± 0.34 for Tiwi Island population (Northern Territory) (Table 2). A similar pattern was observed for percentage of polymorphic loci (PPL), which ranged from 62 to 87% for Nickol Bay and Tiwi Island populations, respectively. Contrastingly, average private allelic richness (A_PR_) and average MAF of polymorphic loci were slightly higher for Western Australia (0.03 ± 0.14 and 0.18) than for both northern Queensland (Bramston Beach, Etty Bay, and Townville; 0.004 ± 0.039 and 0.13) and Northern Territory (Gulf of Carpentaria, Joseph Bonaparte Gulf, and Tiwi Island; 0.03 ± 0.13 and 0.15) populations, respectively.

**Table 2:** Genetic diversity indices for the *Penaeus monodon* populations sampled

Population	N	A_**R**_ (± SD)	A_**PR**_ (± SD)	PPL (Av. MAF)	H_**o**_ (± SD)	H_**E**_ (± SD)	Av. MLH (± SD)	F_**IS**_ (***p*** < 0.05)	N_**eLD**_[95% C.I.]
Bramston Beach (BB)	60	1.66 ± 0.39	0.004 ± 0.039	0.79 (0.13)	0.13 ± 0.15	0.15 ± 0.16	0.13 ± 0.01	0.12	24,121.6 [11,024.4- ∞]
Etty Bay (EB)	50	1.66 ± 0.40	0.01 ± 0.05	0.77 (0.14)	0.13 ± 0.15	0.15 ± 0.16	0.13 ± 0.02	0.14	147.5 [146–149]
Townsville (TSV)	22	1.68 ± 0.45	0.03 ± 0.13	0.69 (0.15)	0.13 ± 0.16	0.15 ± 0.17	0.13 ± 0.004	0.10	2504.6 [17,897.9 – 4175.2]
Gulf of Carpentaria (GC)	33	1.74 ± 0.39	0.01 ± 0.07	0.80 (0.15)	0.15 ± 0.15	0.17 ± 0.17	0.15 ± 0.02	0.13	1427.4 [1263.1 –1640.5]
Joseph Bonaparte Gulf (JBG)	34	1.74 ± 0.38	0.01 ± 0.06	0.81 (0.14)	0.15 ± 0.16	0.17 ± 0.17	0.15 ± 0.004	0.11	7065.4 [4454.1 – 15,832]
Tiwi Island (TIW)	56	1.75 ± 0.34	0.01 ± 0.06	0.87 (0.13)	0.15 ± 0.15	0.17 ± 0.16	0.15 ± 0.004	0.12	8125.2 [5853.9 – 13,264.8]
Nickol Bay (NKB)	23	1.61 ± 0.48	0.03 ± 0.14	0.62 (0.18)	0.16 ± 0.20	0.16 ± 0.18	0.16 ± 0.04	− 0.04	165.4 [159.9–171.2]

The parameters calculated number of samples (N), mean allelic richness values (A_R_), private allelic richness (A_PR_), percentage of polymorphic loci (PPL, average MAF of polymorphic loci), mean observed heterozygosity (H_O_), average expected heterozygosity (H_E_), average multi-locus heterozygosity (Av. MLH), inbreeding coefficient (*F*_*IS*_), and effective population size (N_eLD_)

Average observed heterozygosity (H_O_) across all seven populations ranged from 0.13 ± 0.15 to 0.16 ± 0.2 and average expected heterozygosity (H_E_) ranged from 0.15 ± 0.16 to 0.17 ± 0.17 while average multi-locus heterozygosity (Av. MLH) within populations ranged from 0.13 ± 0.01 to 0.16 ± 0.04 (Table 2). Among populations, Western Australia displayed the highest Ho and Av. MLH values (0.16 ± 0.2 and 0.16 ± 0.04, respectively) whereas northern Queensland showed the lowest levels (0.13 ± 0.15 and 0.13 ± 0.01, respectively). Moreover, average H_O_ values were lower compared to average H_E_ values in all northern Queensland and Northern Territory populations with the exception of Western Australia where average H_O_ and H_E_ values were similar (Table 2). Average *F*_*IS*_ values for both northern Queensland and Northern Territory sites were positive and ranged from 0.10 to 0.14, while average *F*_*IS*_ value for Western Australia population was negative, but close to zero (− 0.04). Estimated N_eLD_ varied across populations and ranged from 165.4 in Western Australia (95% CI = 159.9–171.2) to 24,121 in Bramston Beach (95% CI = 11,024 - ∞), with Western Australia and Etty Bay populations having the lowest N_eLD_ across all seven populations (Table 2).

### Population differentiation and genetic structure

**Fig. 1:**
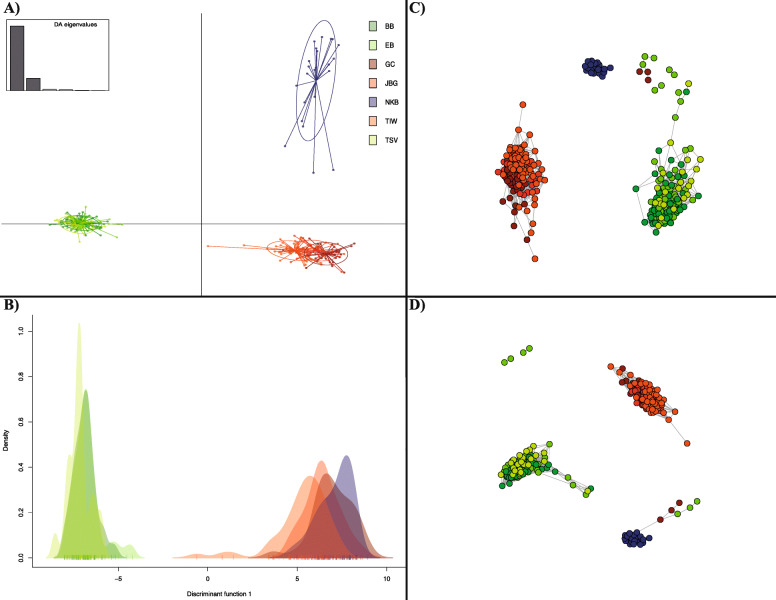
Population structure of 278 individuals of *Penaeus monodon* samples using 10,624 SNPs (BB: Bramston Beach, EB: Etty Bay, Townsville: TSV, Gulf of Carpentaria: GC, Joseph Bonaparte Gulf: JBG, Tiwi Island: TIW, and Nickol Bay: NKB). **a** Discriminant Analysis of Principal Components (DAPC) scatterplot and **b** an individual density plot on the first discriminant function created through the R package ‘*adegenet* ‘; and **c** and **d** Population networks constructed using the Netview P v.1.0 pipeline at kNN = 30 and 60. Dots represent individuals, whereas colored ellipses correspond to sampling origin

DAPC and NETVIEW analyses (see Methods and Additional file 1) suggested regional structure among Australian *P. monodon* populations, with evidence for three clusters: a north Queensland group (Bramston Beach, Etty Bay, and Townville), a Northern Territory group (Gulf of Carpentaria, Joseph Bonaparte Gulf, and Tiwi Island), and Western Australia (Nickol Bay) (Fig. 1). The individual density distribution of the first retained discriminant function indicated separation of north Queensland shrimp from those in the Northern Territory and Western Australia groups (Fig. 1b). Fine-scale population network analysis using *Netview R* provided greater resolution at the individual level between populations and demonstrated the same three genetic clusters at k-NN = 60 (Fig. 1c and d).

Across all *P. monodon* populations pairwise *F*_*ST*_ values based on unbiased [35] ranged from 0.001 to 0.107, while pairwise standard genetic distances [36] similarly ranged from 0.002 to 0.028 (Table 3). All pairwise *F*_*ST*_ comparisons were significant (*p* ≤ 0.05) except for pairwise comparison between Townsville and Etty Bay populations in north Queensland. Not surprisingly, the most geographically separated sites (Nickol Bay in Western Australia and three north Queensland populations at approx. 3091 to 3137 km apart) exhibited the largest significant pairwise *F*_*ST*_ (0.107) and standard genetic distance (0.028) values (Table 4). Subsequent regression-based analysis also demonstrated a significant linear relationship between pairwise geographic distance and pairwise genetic distance among all sampled populations (r^2^ = 0.86, *p* = 0.006; see Additional file 3).

**Table 3:** Population differentiation estimates for *Penaeus monodon* populations sampled

	Bramston Beach	Etty Bay	Townsville	Gulf of Carpentaria	Joseph Bonaparte Gulf	Tiwi Island	Nickol Bay
Bramston Beach		0.002	0.003	0.010	0.009	0.008	0.026
Etty Bay	0.001		0.004	0.011	0.010	0.008	0.026
Townsville	0.001	**0.001**		0.012	0.011	0.009	0.028
Gulf of Carpentaria	0.039	0.039	0.037		0.004	0.004	0.020
Joseph Bonaparte Gulf	0.034	0.034	0.033	0.002		0.003	0.020
Tiwi Island	0.028	0.028	0.026	0.003	0.001		0.019
Nickol Bay	0.107	0.107	0.107	0.071	0.069	0.069	

Population pairwise *F*_*ST*_ [35] and Nei’s genetic distance [36] estimates computed by using the R package StAMPP v.1.5.1 [37]. Pairwise *F*_*ST*_ values are shown below the diagonal, Nei’s genetic distance are reported above. All *F*_*ST*_ values were significant at p ≤ 0.05 following 1000 bootstraps performed across loci to generate confidence intervals. The only non-significant *F*_*ST*_ value (*p* > 0.05) is in bold type

**Table 4:** Analysis of molecular variance (AMOVA) of *Penaeus monodon* from seven Australian locations (groups were assigned from DAPC analysis (see Fig. 1))

Variance partition	d.f.	Sum of squares	Variance component	% of variation	Fixation indexes	***p***-value
Among groups	2	6973.99	18.84	4.21	FCT: 0.042	< 0.01
Among populations within groups	4	1999.07	0.89	0.20	FSC: 0.002	< 0.01
Within populations	549	234,808.63	427.70	95.59	FST: 0.044	< 0.01
Total	555	243,780.68	447.43			

*d.f.* Degrees of freedom

AMOVA analysis also grouped populations by geographical region: north Queensland (Bramston Beach, Etty Bay, and Townsville), Northern Territory (Gulf of Carpentaria, Joseph Bonaparte Gulf, and Tiwi Island), and Western Australia (Nickol Bay). Hierarchical population genetic analyses indicated majority of variation (95.6%) being explained among individuals within populations (*p* <  0.01; Table 4). Differences among geographically discrete populations accounted for only 4.2% of the total variance (*p* <  0.01), while 0.2% of the total variance (*p* <  0.01) was explained by variation among populations within each group.

### Detection of outlier loci

**Fig. 2:**
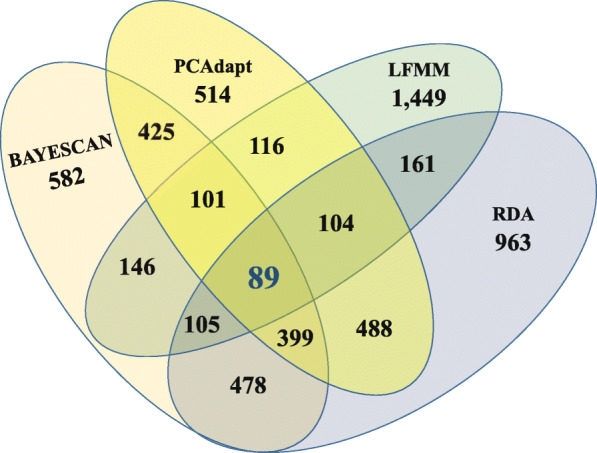
Venn diagram showing the total number of putative SNP significantly associated with at least one environmental variable. The total number of SNP is reported in each panel. The 89 outlier SNPs on the intersections (shaded area) were retained for further analysis

Initial Bayescan v.2.1 and *PCAdapt* analyses (FDR = 0.01 for both; see Methods) detected 582 and 514 outlier SNPs, respectively, among the 10,624 SNPs retained for each individual (see Additional file 1). LFMM analysis (K = 3 and FDR = 0.05; see Methods) identified 1449 outlier SNPs that were significantly associated with at least one of seven environmental parameters tested. Full RDA model supported the role of environmental variables in shaping the distribution of SNP genotypes (*p* < 0.01; R^2^ = 0.048; adjusted R^2^ = 0.031) with 43.5 and 30.2% of inherent genetic variation explained by the first and second RDA axes, respectively. Based on these two significantly constrained axes the RDA model collectively yielded 963 candidate outlier SNPs. Lastly, the rigorous evaluation of both PD and two EA analyses (see Methods) identified 425 and 161 outlier SNPs respectively, of which 89 (21 and 55%) overlapped (see Fig. 2 and Additional file 4), leaving 10,535 SNPs as neutral (see Additional file 5). Both outlier and neutral SNP were subjected to further analyses (see Additional file 1).

### Genetic structure based on outlier and neutral markers

**Fig. 3:**
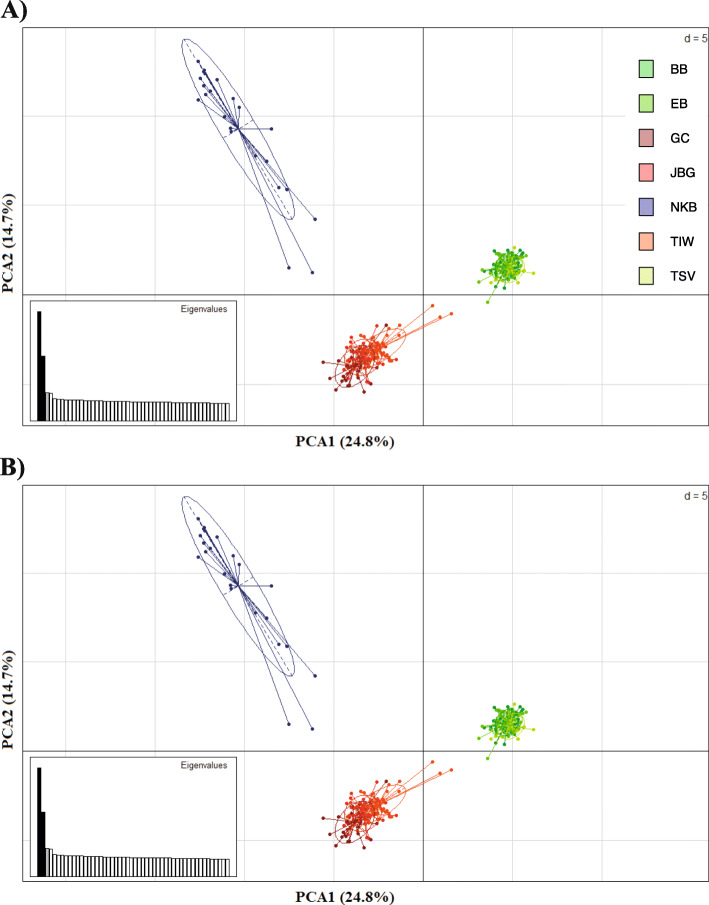
Principal components analysis based allele frequencies for (**a**) 10,535 neutral SNPs loci and (**b**) 89 outlier SNPs loci (BB: Bramston Beach, EB: Etty Bay, Townsville: TSV, Gulf of Carpentaria: GC, Joseph Bonaparte Gulf: JBG, Tiwi Island: TIW, and Nickol Bay: NKB)

PCA analysis of all individuals for both neutral and outlier SNP separated Australian populations into the same three clusters as DAPC and Netview analyses (Western Australia separate from north Queensland and Northern Territory populations; see above). Considering the 10,535 neutral SNPs, PC1 and PC2 explained 24.8 and 14.7% of the total genetic variance, respectively (Fig. 3a). Considering the 89 outlier SNPs, PC1 and PC2 explained 5.8 and 1.0% of the total genetic variance, respectively (Fig. 3b).

Pairwise *F*_*ST*_ estimates between populations differed depending on assessment of neutral or outlier loci (see Additional file 6). Genetic differentiation was significant (*p* < 0.05) for all pairwise comparisons when 10,535 neutral SNPs were assessed. Pairwise *F*_*ST*_ values ranged from 0.001 (Bramston Beach vs. Etty Bay and Joseph Bonaparte Gulf vs. Tiwi Island) to 0.1 (northern Queensland populations vs. Western Australia population). In a similar way, using 89 outlier loci, *F*_*ST*_ values between north Queensland populations and Western Australia (*F*_*ST*_ = 0.60–0.64) were in all cases higher than those between Northern Territory populations and Western Australia (*F*_*ST*_ = 0.19–0.22; *p* ≤ 0.01). *F*_*ST*_ values differed significantly between all populations within the Northern Territory (*p* ≤ 0. 04); however, no *F*_*ST*_ values differed significantly between populations within northern Queensland (*p* > 0.4).

### Environmental factors association with genetic variation

**Fig. 4:**
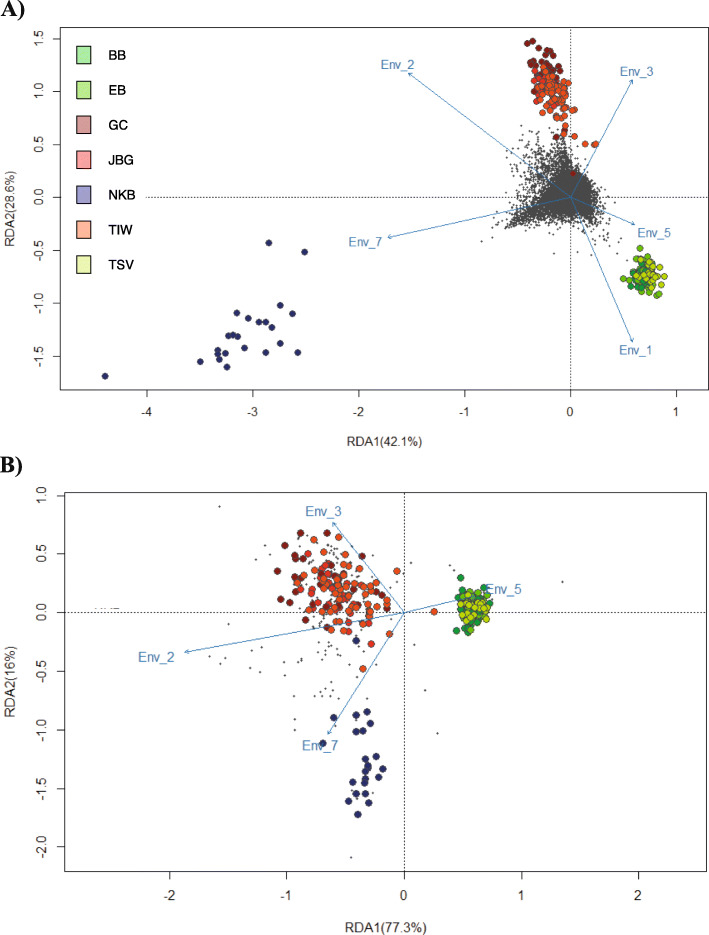
Redundancy analysis on (**a**) 10,535 neutral loci and (**b**) 89 outlier SNPs allele frequencies in seven populations of *Penaeus monodon* in Australia. Explanatory variables (arrows) were RDA axes retained as important variable selection accounting for genetic variation (Env_2: surface temperature maximum, Env_3: surface temperature minimum, Env_5: surface phytoplankton mean, Env_7: benthic current velocity mean, and Env_1: surface salinity mean)

ANOVA for RDA analyses demonstrated that five (Env_1, Env_2, Env_3, Env_5, and Env_7) and four (Env_2, Env_3, Env_5, Env_7) environmental factors (see Methods) were significantly associated with genetic variation in identified neutral and outlier SNP datasets (*p* = 0.001 and 0.001, adjusted R^2^ = 0.03 and 0.2), respectively (Fig. 4). When partitioning the relative importance of geographic proximity among sampled sites using partial RDA, the most important environmental predictors for neutral SNP were ranked as: Env_2 (F = 1.7, *p* = 0.001) > Env_1 (F = 1.5, *p* = 0.001) > Env_3 (F = 1.2, *p* = 0.001). For outlier SNP, Env_2 and Env_3 were both significant for putative adaptive genetic variation (*p* = 0.001); however, Env_2 explained 13% of inherent genetic variation while Env_3 explained 2.8%. When both neutral and outlier SNP datasets were combined, surface temperature max and min (Env_2 and Env_3 respectively) were the highest ranked predictors contributing to the genetic variation of Australian *P. monodon*. Variance partitioning based on neutral and outlier loci RDA models revealed that environmental effects explained 4.3 and 20.2% of genetic structure variation, while geographic location explained 12.9 and 2.8%, respectively. The remaining genetic structure variance was explained by the combination of environmental effect and geographic location.

MRM correlation analysis conducted on geographic distance and pairwise population linearized *F*_*ST*_ values was significant (r^2^ = 0.9, *p* = 0.008). MRM models including surface temperature maximum (Env_2) and surface temperature minimum (Env_3) exhibited significant relationships with pairwise *F*_*ST*_ values (*p* = 0.001 and 0.008, adjusted R^2^ = 0.95 and 0.90), respectively (Table 5). Moreover, MRM models explained a large and significant proportion of genetic variability (> 90%), indicating that these environmental variables and geographic distance are significantly correlated and thus potentially constrain gene flow synergistically.

**Table 5:** Results of multiple regression of distance matrices (MRM) tests of geographic distance, genetic differentiation (*F*_*ST*_) of 89 outlier loci, and significant environmental variables between *Penaeus monodon* populations in Australia

MRM models	Adj-R^**2**^	***p***-value
Distances ~ *F*_*ST*_ + surface temperature max	0.95	0.001
Distances ~ *F*_*ST*_ + surface temperature min	0.90	0.008
Distances ~ *F*_*ST*_ + surface temperature max + surface temperature min	0.95	0.002

### Gene ontology

Among the 89 SNPs that were found associated with environmental variables, 11 (12.4%) yielded matches to 173 total contigs in the *P. monodon* transcriptome with E value ≤1e-5 (see Additional file 7). Three of these 11 outlier SNPs exhibited BLAST hits in only one allele (ID: PM_10591, PM2065, and PM10621; see Additional file 7) and were thus excluded from translated protein comparison. Protein translation of the remaining eight outlier SNPs demonstrated the following: 1) five contained synonymous and non-synonymous mutations in two and four reading frames, 2) two contained synonymous and non-synonymous mutations in one and five reading frames, and 3) one contained synonymous and non-synonymous mutations in zero and six reading frames, respectively (see Additional file 8). However, only two of these eight outlier SNPs exhibited 100% pairwise identity with > 90% query cover to contigs within the *P. monodon* transcriptome (PM1958 and PM4714). More specifically, SNP PM1958 matched “calcium-activated chloride channel regulator”, “calcium-activated chloride channel regulator 2-like”, and “epithelial chloride channel-like” contigs while SNP PM4714 matched “uncharacterized protein APZ42_034504” or “Predicted: uncharacterized protein LOC108744461 isoform X4” contigs (Table 6). The remaining majority of outlier SNP (*n* = 78 or 87.6%) did not exhibit sufficiently strong matches to any contigs within the *P. monodon* transcriptome and were thus concluded to reside in non-coding (i.e., absent) genomic regions.

**Table 6:** Characterization of high-quality BLAST matches obtained in comparison with the transcriptome *Penaeus monodon* database

	PM1958	PM4714
**Functional Annotation**	Calcium-activated chloride channel regulator Calcium-activated chloride channel regulator 2-like Epithelial chloride channel -like	PREDICTED: uncharacterized protein LOC108744461 isoform X4 Uncharacterized protein APZ42_034504
**E-value**	2.01E-29	3.36E-27
**% Identity**	100	94.2
**Bit-Score**	128.5	121.2
**Translation Frame 1**	CRRVRRLP**V**PLPPRLQPRGAAPI CRRVRRLP**I**PLPPRLQPRGAAPI	CST*GTASWTGTRGRTCSTRSRD CST**S**GTASWTGTRGRTCSTRSRD
**Translation Frame 2**	ADAYAAYPYPYHPGYSHEELPP ADAYAAYPYPYHPGYSHEELPP	AVH**K**AQRAGRALAAALVAHEAE AVH**Q**AQRAGRALAAALVAHEAE
**Translation Frame 3**	QTRTPPT**R**TPTTQATATRSCPH QTRTPPT**H**TPTTQATATRSCPH	QYIRHSELDGHSRPHL*HTKPR QYIRHSELDGHSRPHL*HTKPR

Bold characters indicate SNP location within translated protein sequences. Asterisks (*) indicate stop codons

## Discussion

The giant black tiger shrimp (*P. monodon*) is an important aquaculture species in Australia; however, to date only limited information is available regarding the influence of environmental pressures on the genetic structure of geographically discrete populations. Based on results from this study, three distinct genetic groups (i.e., stocks) were revealed across the geographic distribution range of Australian *P. monodon*: north Queensland (Bramston Beach, Etty Bay, and Townville), Northern Territory (Gulf of Carpentaria, Joseph Bonaparte Gulf, and Tiwi Island), and Western Australia (Nickol Bay). Of note is that the Western Australia population exhibited the lowest level of genetic variation of all assessed populations, which could be due to restricted gene flow between Western Australia and Northern Territory populations. Using multivariate analyses that considered both geographic distance and environmental factors it was determined that the majority of SNPs are neutral (*n* = 10,535), while a small subset of outliers are putatively adaptive (*n* = 89). Surface temperature maximum and minimum provided the strongest correlative explanation for the presence of these outliers (see below).

### Population genetic diversity

Genetic diversity and stock assessments of Australian *P. monodon* were undertaken using all 10,624 SNP (i.e., neutral and outliers combined). Relative reduced genetic diversity (H_O_, H_E_, and Av.MLH) was observed for Australian *P. monodon* compared to other recently assessed crustacean species. More specifically, European green crab (*Carcinus maenas*) exhibited average H_O_ and H_E_ of 0.254 and 0.256 [38] while European lobster (*Homarus gammarus*) had H_O_ and H_E_ that ranged between 0.049–0.63 and 0.179–0.504, respectively [39] and scalloped spiny lobster (*Panulirus homarus*) had H_O_, H_E_, and Av.MLH that ranged between 0.166–0.184, 0.226–0.233, and 0.168–0.186, respectively [40]. The small reduction in genetic diversity observed for Australian *P. monodon* could be due to technical artefacts of the RADseq-based genotyping (i.e., null alleles) [41, 42] and/or sampling bias (i.e., Wahlund effect) [43, 44]. However, H_O_ deficiency has also been observed in Bangladesh *P. monodon* using 10 microsatellite markers and 14 SNP loci [45], so the exact cause is yet to be fully resolved, but is more likely technical than biological.

In the Western Australia (Nickol Bay) population, heterozygosity (i.e., H_O_ and H_E_) were equal or higher than other populations despite A_R_ and PPL being slightly lower (0.01–0.02) (see Results). This slight reduction in A_R_ and PPL is most likely due to the smaller sample size in our study for the Western Australia population [46]. SNP genotyping of this Western Australia population revealed equal or higher heterozygosity, despite previous allozyme and microsatellite based demonstrations of reduced heterozygosity and number of alleles in *P. monodon* from the same Western Australia region [31, 47]. These previous *P. monodon* population genetics studies concluded that observed reductions in heterozygosity and number of alleles were most likely driven by founder effects or bottleneck events. As such, based on the entire Western Australia SNP dataset encompassing 10,624 genome-wide SNPs, Western Australia *P. monodon* appears from our dataset to not have undergone a bottleneck (i.e., heterozygosity not reduced), but rather has retained similar heterozygosity to Northern Territory and north Queensland populations. Differences between our dataset and those using microsatellites, allozymes and mtDNA may simply be due to the level of genetic resolution of these markers when comparing allelic diversity, where our high-resolution sampling captured more of the true genetic diversity within the *P. monodon* genome. Likewise, Lemopoulos et al. [48] demonstrated that SNPs were more informative than microsatellites for applications that required individual-level genotype information (i.e., estimating relatedness and genetic diversity with low sample sizes from small populations). North Queensland and Northern Territory *P. monodon* populations exhibited F_*IS*_ values > 10%, which exceeded a general F_*IS*_ threshold of ~ 10% suggested by Moss et al. [49] in a study of the effects of inbreeding on survival and growth of Pacific white shrimp (*Litopenaeus vannamei*). However, slightly positive F_*IS*_ values observed here are most likely a result of DArTSeq genotyping technical artifacts (ie., null alleles) rather than of biological origin. Furthermore, the large N_eLD_ estimates determined for all Australian *P. monodon* populations are likely to reduce any possible effect of inbreeding [50]. It is also noteworthy, that F_*IS*_ values in this study are lower than observed in wild Pacific Ocean *L. vannamei* collected from Panama to Mexico (F_*IS*_ = 0.53) [51] and along the Mexican coast (F_*IS*_ = 0.36) [52].

### Population differentiation and genetic structure

Pairwise *F*_*ST*_ analysis for north Queensland and Northern Territory Australian *P. monodon* populations revealed relatively weak genetic structuring (0–0.039), except for Western Australia (0.069–0.107), which had the highest levels detected (Table 3). Additionally, genetic differentiation increased proportionally with geographic distance and followed the IBD pattern across the entire sampled range, which is in agreement with previous observations based on mtDNA and microsatellite sequences [25, 31, 33]. However, this finding contrasts the *F*_*ST*_ values observed between Western Australia and Northern Territory (0.116) and Queensland (0.032) [34]. As such, the significantly different genetic composition between Western Australia and all other populations may reflect the effects of restricted gene flow and genetic drift, which is not surprising for *P. monodon* given the relatively short offshore planktonic larval phase (approximately 20 days), during which larvae and post-larvae disperse and migrate to nursery areas (e.g., in-shore areas and mangrove estuaries) [27]. Moreover, the evidence for adaptive divergence among Australian *P. monodon* populations presented here (see below) suggests that divergent selection may be contributing to genetic divergence despite genetic drift [53]. Regardless of the exact cause, these results suggest that there is a restriction in gene flow between geographically disparate Australian *P. monodon* populations.

Assessment of population structure using *F*_*ST*_, DAPC and Netview analyses revealed regional structure in Australian *P. monodon* with three major population groupings: north Queensland (Bramston Beach, Etty Bay, and Townsville), Northern Territory (Gulf of Carpentaria, Joseph Bonaparte Gulf, and Tiwi Island), and Western Australia (Nickol Bay) (Fig. 1). One possible explanation for this genetic structure is the presence of biogeographic barriers between eastern, northern, and Western Australia that caused restricted gene flow between *P. monodon* populations. Upwelling of deep cold water along the north-western Australian coastline, which has existed since the Late Miocene period, is one biogeographical barrier known to prevent gene flow between Western Australia and other Australian regions [54]. Moreover, repeated declines in sea surface levels of 100–140 m during the Pleistocene caused biogeographic isolation between northern and eastern Australia due to repeated emergence of land bridges between northern Australia, Torres Strait, and New Guinea [55–58]. Similar patterns in genetic structure between Australian populations have been observed for other marine species such as Australian Spanish mackerel (*Scomberomorus commerson*) [59], mud crab (*Scylla serrata*) [60], brown tiger prawn (*Penaeus esculentus*) [61], and barramundi (*Lates calcarifer*) [62]. Previous *P. monodon* genetic differentiation investigations based on allozyme, mitochondrial DNA and microsatellite markers also revealed significant genetic partitioning among Australian and south-east Asian *P. monodon* populations indicating significant bio-geographic barriers to dispersal [25, 31, 33, 34]. Accordingly, the current observed genetic differentiation among Australian *P. monodon* populations appear to be driven by the presence of biogeographic barriers between eastern, northern, and Western Australia that effectively limited or prevented gene flow over evolutionary timescales.

### Evidence for local adaptation

PD and EA analyses collectively identified 89 overlapping outlier SNPs, which accounted for 21 and 55% of total outlier SNPs detected, respectively (Fig. 2). This observation is in agreement with previous studies that also found EA approaches to perform better than PD approaches for detection of loci under putative divergent selection [10, 20]. Moreover, the observed levels of genetic structure based on these 89 outlier SNPs agreed with the genetic structure observed when all 10,535 neutral SNPs were considered.

Analysis of population structure based on 89 outlier SNPs using PCA presented a similar spatial pattern as to PCA based on 10,535 neutral SNPs in the form of three groups: north Queensland, Northern Territory, and Western Australia (Nickol Bay). Several causes may contribute to the same population structure being determined when either neutral or outlier loci were used (e.g., life history, natural selection, and environmental heterogeneity [63–65]; however, determination of the exact cause requires further investigation. Our findings agree with previous SNP-based studies conducted on other species with a planktonic larval phase (e.g., Eulachon (*Thaleichthys pacificus*)*,* European green crab (*C. maenas*), and Atlantic salmon (*Salmo salar*)) that were determined to have low level gene flow and genome-wide divergence [38, 64, 66], respectively. These outlier-based analyses suggest that key environmental factors (see Results) and geographic distance, synergistically or independently, contributed to the generation of the 89 outlier SNPs observed among Australian *P. monodon* populations (i.e., adaptive divergence drivers). This conclusion is supported by other studies that also demonstrated that association between environmental variables and outlier SNPs could be indicative of local adaptation [9, 10, 23, 67, 68].

### Environmental variables contributing to genetic structure

RAD, pRAD and MRM analyses demonstrated a strong relationship between geographic distance, environmental distance, and genetic differentiation in both neutral (*n* = 10,535) and outlier (*n* = 89) SNPs inherent to wild Australian *P. monodon*. This significant positive correlation suggests that: 1) gene flow between sampled sites may be impacted by natural selection or 2) geographically discrete Australian *P. monodon* populations have adapted to region-specific environmental variables (e.g., temperature). Geographic isolation due to biogeographic barriers (see above) could have caused initial level of genetic differentiation between *P. monodon* populations with subsequent divergent selection imposed by environmental factors leading to increased differentiation across evolutionary time. This interpretation is supported by RDA analysis, which indicated that the effects of surface temperature maximum and minimum (Env_2 and Env_3 respectively) were more pronounced than geographic distance for both neutral and outlier SNP.

RAD and partial RAD analyses conducted on outlier SNP demonstrated that surface temperature maximum (Env_2) explained five and six times more genetic variation than was explained by surface temperature minimum (Env_3), respectively. Therefore, it is plausible that surface temperature maximum (Env_2) could be the predominant driver of local adaption among Australian *P. monodon* populations. In a review of local adaptation among populations of marine invertebrates, Sanford and Kelly [69] found that approximately 44% of surveyed studies provided evidence of local adaptation to temperature. Moreover, several studies targeting marine invertebrates demonstrated the role of sea surface temperature in shaping genetic differences among populations (e.g., American lobster (*H. americanus*) [10], European green crab (*C. maenas*) [68], eastern oyster (*C. virginica*) [9], sea scallop (*Placopecten magellanicus*) [67], and giant California sea cucumber (*Parastichopus californicus*) [23]). Winter sea surface temperature was also demonstrated to be the most likely driver of local adaptation and limiter of gene flow among North American populations of *C. maenas* [68]. In Australia, sea surface temperatures have become significantly warmer between 1950 and 2007 along north-eastern and north-western tropical coasts by 0.12 °C and 0.11 °C per decade, respectively [70], while sea surface temperature in south-western Australia has risen by 0.026 to 0.034 °C per year between 1985 and 2004 [71]. Moreover, surface temperature maximum and minimums occur during summer and winter, respectively, which is when *P. monodon* broodstock emigrate out of estuaries into foreshores for spawning and, thus, extreme swings could influence individual fitness (i.e., breeding success). Further studies are needed to elucidate the existence and extent of population-specific thermal adaptation among Australia *P. monodon* populations and the potential functional genomics implications of the identified 89 outlier SNPs.

### Highlights from gene ontology

The combination of PD and EA approaches identified 89 outlier SNPs that are potential targets for local adaptation within the Australian *P. monodon* genome. Only 11 of these 89 outlier SNPs matched *P. monodon* transcriptome contigs [72] with subsequent protein translation of eight outlier SNPs demonstrating more non-synonymous changes than synonymous changes across all six reading frames. Despite the greater likelihood that non-synonymous mutations will have functional implications [73, 74], all 11 of these outlier SNPs with *P. monodon* transcriptome matches should be considered as candidates for future research into divergent selection driven local adaptation among these geographically discrete populations [75].

Of these 11 outlier SNPs, one matched a contig annotated as “calcium-activated chloride channel regulator” (*CLCA*) (Table 6). *CLCA* is involved in cellular physiology functions such as neuronal and cardiac action, muscle contraction, and epithelial secretion [76–78]. In Pacific white shrimp *L. vannamei*, a similar calcium-activated chloride channel gene was shown to be expressed in gill cells and exhibit potential involvement in osmoregulation because of observed response to salinity challenge [79]. As such, future *P. monodon* gene-by-environment studies should consider investigating the potential role of this gene in local adaptation to population-specific environmental conditions.

The remaining 78 outlier SNPs are presumed to be located in non-coding regions absent from the transcriptome, or in genes with low-level expression that were not captured within initial transcriptome assembly [80–82].

### Implications for aquaculture management and future research directions

The black tiger shrimp is an important aquaculture and fishery species in Australia. Therefore, the identification of genetic stock units among wild populations is crucial for fishery and aquaculture broodstock management. Neutral and adaptive population structure findings suggested that Australian *P. monodon* should be managed as three separate stocks: north Queensland, Northern Territory, and Western Australia. The levels of genetic diversity revealed in the present study using both neutral and outlier SNP are useful for aquaculture purposes such as selective breeding programs, maintaining stock diversity, and distinguishing hatchery stocks from wild populations. Moreover, in addition to reduced gene flow, thermal adaptation is also likely to contribute to a greater divergence in the Western Australia population. Regardless, the strong genetic structure and presence of rare alleles in all seven *P. monodon* populations suggests that these populations should be managed accordingly to maintain genetic integrity. Environmental factors can influence genetic diversity and population structure in marine species and an accurate understanding of this influence can both improve fisheries management and help predict responses to environmental change [67]. This study suggests that both geographical distance and environmental factors interact to influence the genetic structure of Australian *P. monodon*; however, the magnitude of influence for each of these factors is hard to determine conclusively. This study also provides evidence for environmentally-driven selection pressure on geographically discrete populations, which can be utilized to help ensure sustainable management of Australian *P. monodon* (e.g., guidance for future re-establishment of populations inhabiting similar thermal gradients). Practically, a population that is potentially under local adaptive pressures may be an important source of private or rare alleles that can enhance population resistance to future environmental change (e.g., naturally or via selective breeding programs) or assist natural migration [83, 84].

## Conclusions

We utilized a SNP dataset containing 10,624 loci to determine genetic population structure and local adaptation across seven populations of Australian black tiger prawn *P. monodon* (*n* = 278 individuals). This study provides novel insights that assist the development and implementation of *P. monodon* aquaculture and fishery management practices within Australia. Analysis of population structure using both neutral (*n* = 10,535) and outlier (*n* = 89) SNPs suggest that Australian *P. monodon* should be managed as three separate stocks (north Queensland, Northern Territory, and Western Australia) and that geographically discrete *P. monodon* populations have likely undergone local adaptation to region-specific thermal regimes. Future studies should investigate the role that outlier SNPs potentially play in local adaptation in order to advance wild stock structure preservation and help facilitate selective breeding programs.

## Methods

### Sample collection and DNA extraction

**Fig. 5:**
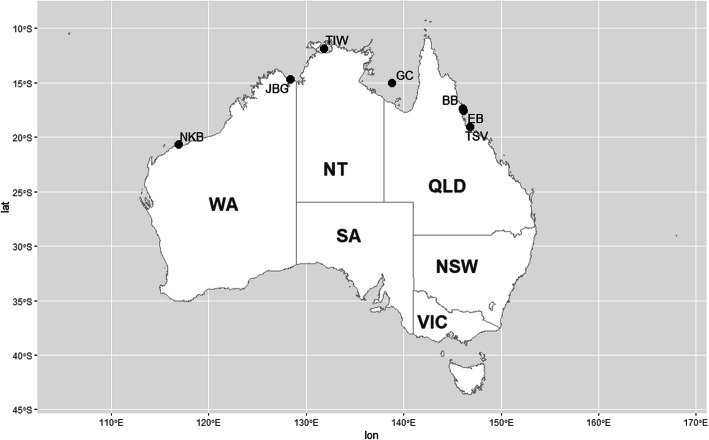
Map showing the seven localities where 283 wild *Penaeus monodon* samples were collected (BB: Bramston Beach, EB: Etty Bay, Townsville: TSV, Gulf of Carpentaria: GC, Joseph Bonaparte Gulf: JBG, Tiwi Island: TIW, and Nickol Bay: NKB). The map was created using R package ‘*ggplot2*’ version 3.2.1 (https://github.com/tidyverse/ggplot2) [85] and R package *‘ozmaps*’ version 0.3.6 (https://github.com/mdsumner/ozmaps) [86]

Individual *P. monodon* (*n* = 283) were collected from seven locations around Australia between 2015 and 2017 (Fig. 5). More specifically, wild adult *P. monodon* were obtained by trawling from Bramston Beach (*n* = 60, BB), Etty Bay (*n* = 50, EB), Townsville (*n* = 22, TSV), Joseph Bonaparte Gulf (*n* = 34, JBG), Tiwi Island (*n* = 56, TIW), Gulf of Carpentaria (*n* = 35, GC), and Nickol Bay (*n* = 26, NKB) (see Additional file 9). Pleopod tissue samples were collected from each adult individual using pre-sterilised scissors followed by preservation in 80% ethanol and − 20 °C storage until DNA extraction.

Genomic DNA (gDNA) was extracted from each pleopod tissue sample following a modified cetyl trimethyl ammonium bromide (CTAB) protocol [44, 87]. Briefly, 100 mg preserved tissue was digested in a CTAB buffer with 20 mg/mL proteinase K for ≥6 h at 55 °C under gentle agitation followed by one phenol–chloroform–isoamyl alcohol phase separation, two chloroform – isoamyl alcohol phase separations, overnight isopropanol precipitation (4 °C), two 70% ethanol washes, and elution in Tris-EDTA buffer [88]. All gDNA extractions were subsequently cleaned using Sephadex™ G-50 [89]. Extracted gDNA samples were assessed for quantity and quality using Nanodrop 1000 Spectrophotometer (260:230 nm and 260:280 nm ratios; ThermoFisher Scientific Pty Ltd., Australia) and 0.8% agarose gel containing GelGreen (ThermoFisher Scientific Pty Ltd., Australia). All gDNA samples were then diluted with Tris-EDTA buffer to a normalized final concentration of 50 ng/μl before shipping to Diversity Arrays Technology Sequencing (DArTseq™; Canberra Australia) for library preparation and DArTseq™ sequencing.

### SNP genotyping and quality control

SNP discovery and genotyping were performed by Diversity Arrays Technology using DArTseq™ hybridization-based sequencing technology on next-generation sequencing (NGS) platforms as per [87, 90, 91]. DArTSeq™ sequences were further filtered using custom *dartQC* python scripts (available at https://github.com/esteinig/dartqc). More specifically, SNP sequences matching the following criteria were removed from the dataset (see Additional file 1): (1) average read depth < 7, (2) average repeatability < 90%, (3) call rate < 80%, (4) similar sequence clusters > 0.95, and (5) minor allele frequency (MAF) < 0.02.

All individuals and remaining loci were subsequently tested for missing data and linkage disequilibrium (LD) using *PLINK 1.9* [92]. All individuals with high rate of missing data (> 40%) were excluded from the dataset. From the pairs of loci determined to be in LD (with a Pearson coefficient of determination (r^2^) threshold of 0.2), SNPs with the lowest call rate value were removed. Lastly, remaining SNP dataset for each population was then assessed for deviation from Hardy–Weinberg equilibrium (HWE) using R package *dartR* [93] and all SNP that significantly deviated from HWE (*p* < 0.0001) were removed.

### Genetic diversity, population differentiation and structure

Several measures were used to estimate population genetic variation and differentiation (see Additional file 1). Observed heterozygosity (H_O_), expected heterozygosity (H_E_), and inbreeding coefficient (*F*_*IS*_) were calculated using the *divBasic* function of R package *diveRsity* version 1.9.90 [94]. *F*_*IS*_ values were determined using 95% confidence intervals and 1000 bootstrap replicates. Allelic richness (A_R_), private allelic richness (A_RA_), and percentage of polymorphic loci (PPL) were calculated for each SNP using rarefaction to avoid sampling size bias in HP-RARE version 1.1 [95]. Multilocus heterozygosity (MLH) was then calculated for all individuals from each population using R package *inbreedR* version 0.3.2 [96]). Effective population size (N_eLD_) was also estimated for each population using *NeEstimator* version 2.1 based on LD and random mating model [97].

Genetic differences among populations and individuals were calculated using R package *StAMPP* version 1.5.1 [37]. Pairwise genetic differentiation values (*F*_*ST*_), along with confidence intervals and probability (*p*) values between populations, were calculated according to Wright [98] and updated by Weir and Cockerham [35]. Nei [36] standard genetic distances (Ds) were calculated across loci in a pairwise comparison between populations and individuals. To evaluate hierarchical population genetic differentiation, an analysis of molecular variance (AMOVA) was implemented using ARLEQUIN version 3.5.2.2 [18]. Statistical significance of each variance component was assessed using 1000 permutations for each of the following hierarchical comparisons: 1) among groups, 2) among populations within groups, and 3) within populations. To determine isolation by distance (IBD), a Mantel test was conducted for the full SNP dataset (10,624) using the *mantel.randtest* function in R package *ade4* [99] with 999 permutations.

To determine the number of genetic groups a Discriminant Analysis of Principal Components (DAPC) was used on individual genotypes in R package *adegenet* version 2.1.1, with number of possible clusters (cluster values (K) of 1–20) determined by running 60 iterations of ‘find.clusters’ function [100]. Bayesian information criterion (BIC) values, which allow choosing the optimal K value, were averaged across all 60 iterations and standard deviation estimated for each K value. Additionally, genotypic relationships between individuals with no prior population assumptions were assessed using R package *NetView* [101, 102]. *NetView* was run through multiple K-nearest neighbour (kNN) values between 1 and 60 to assess both broad and fine scale population structure.

### Environmental data collection and processing

Environmental factors considered ecologically relevant to *P. monodon* [26, 27] were selected from the Bio-ORACLE database (http://www.bio-oracle.org/downloads-to-email.php) [103, 104]. QGIS version 2.18.11 (https://www.qgis.org/) was used to extract values of 23 marine environmental variables (see Additional file 10) based on latitude and longitude of all seven sampling locations around northern Australia (see Fig. 1; Additional file 9). Marine data layers were then produced using monthly averages of climate data from 2000 to 2014 for all seven sampling locations (Fig. 1). Correlation estimates generated for marine data layers and filtered SNP datasets for each population using Pearson Test (R software *cor* and *cor.test* functions) identified seven marine environmental variables with Pearson coefficient of correlation (r) between − 0.75 and 0.75. Marine environmental variables retained for further analysis were: 1) surface salinity mean (Env_1), 2) surface temperature maximum (Env_2), 3) surface temperature minimum (Env_3), 4) surface current velocity mean (Env_4), 5) surface phytoplankton mean (Env_5), 6) benthic temperature mean (Env_6), and 7) benthic current velocity mean (Env_7) (see Additional file 7).

### Detection of loci under selection

The possible effects of divergent selection on the overall pattern of genetic differentiation was assessed by combining the results of two independent population differentiation (PD) and two independent environmental association (EA) approaches (see Additional file 1). First, BayeScan version 2.1 [14, 105] and R package *PCAdap*t [13] were used to identify loci under selection (i.e., two independent PD approaches). Second, to identify environmental variables associated with genetic variation, redundancy analysis (RDA) in the R package *vegan* version 2.5–2 [106, 107] and latent factor mixed models (LFMM) in the R package *LEA* version 2.0.0 [16] were explored (i.e., two independent EA approaches). All four approaches were run on the same SNP dataset using strict parameters (see below). Only candidate outlier SNP identified by all four approaches were retained for subsequent analyses (see below).

BayeScan version 2.1 [14, 105] was run with 20 pilot runs of 5000 iterations followed by 100,000 iterations and an additional burn-in of 50,000 iterations. Alpha levels and *F*_*ST*_ values were ordered from largest to smallest and candidate loci were determined using false discovery rate (FDR) control levels of 0.001, 0.005, 0.01, 0.05, and 0.1 using Bayescan 2.01 function *plot_R.r* [108]. The R package *PCAdap*t [13] with K = 2 and min.maf = 0.05 was used to detect outlier loci based on principal component analysis (PCA) by assuming that markers excessively related with population structure are candidates for local adaptation. The candidate loci were determined using FDR control levels ranging from 0.01 to 0.1.

The linear model redundancy analysis (RDA) was run in R package *vegan* version 2.5.2 [106]. The *vegan* function *ordistep*, which applies a stepwise per mutational ordination method, was used to perform the ‘optimal’ model that corresponded to the highest adjusted coefficient of determination (adj R^2^). To test the significance of the final RDA model, the *vegan* function *anova.cca* was run with 999 permutations then outlier SNPs were identified on each of the first two constrained axes (*p* = 0.001) using the function *outliers*. Finally, LFMM models were run using R package *LEA* version 2.0.0 [16] for each environmental variable with a total number of 10,000 iterations and a burn-in of 5000 iterations. The specified result (K = 3) corresponded with the number of population clusters identified in DAPC from five repetitions. Z-scores for each locus were combined across five replicates and FDR were evaluated using FDR-adjusted *p* values. Candidate loci were determined for each marine environmental variable (*n* = 7; see above) and all candidate loci with FDR-adjusted *p* ≤ 0.05 were retained for subsequent analyses (see below).

### Environmental factors association with genetic variation

To examine adaptive and neutral patterns of variation within SNP obtained across all seven collection locations (Fig. 1), PCA was performed on the retained environmental outlier (*n* = 89) and neutral (*n* = 10,535) loci using R package *adegenet* version 2.1.1 [100]. Retained outlier and neutral loci were then used to calculate genetic differences among populations and individuals using R package *StAMPP* version 1.5.1 [37].

To examine the relative contribution of geographical distances and environmental variables and select which environmental factors best explain Australian *P. monodon* genetic structure, redundancy (RDA) and partial redundancy (pRDA) analyses were conducted for both retained outlier and neutral locus allele frequencies using R package *vegan*. Analyses of variance (ANOVA) with 999 permutations were used to assess the significance of each environmental parameter within the RDA for all RDA and partial pRDA tests. The *vegan* function *ordistep* with 999 permutations was used to evaluate each environmental variable and perform the ‘optimal’ model (i.e., model that best explained genetic structure of each discrete population).

To determine whether isolation by distance (IBD) could explain geographic patterns of differentiation in retained outlier loci (see above), multiple regression on distance matrices (MRM) [109] was conducted in R package *ecodist* [110] with 1000 permutations. Pairwise population *F*_*ST*_ values calculated in *StAMPP* and least-cost geographic distances calculated in *marmap* were used as response values and explanatory variables, respectively, and then MRM models were generated using the marine environmental variables that significantly correlated with genetic variation of retained outlier loci. Note that MRM models focused on the relationship between geographic distance and environmental variables to determine the significance of environment factors on gene flow among populations [68].

### Gene ontology

Each outlier SNP was assessed against an assembled *P. monodon* transcriptome [72] using Geneious Prime version 2019.1.3 [111]. Contigs within assembled *P. monodon* transcriptome that exhibited strong pairwise similarity with any outlier SNP (E-value ≤1e− 5) were compiled and reported along with contig annotations provided by Huerlimann et al. [72]. All SNP that exhibited transcriptome matches were translated into protein sequence using all six reading frames (three and three in 5′ – 3′ and 3′ – 5′, respectively) to assess if mutation caused synonymous or non-synonymous change.

## Supplementary information

**Additional file 1. **SNP data analysis workflow from raw (*n* = 125,511) to final neutral (*n* = 10,535) and outlier (*n* = 89) loci datasets.

**Additional file 2. **Genotypic data. Genotypes of 278 individuals of *P. monodon* at 10,624 adaptive and neutral genome-wide SNPs are included in a standard STRUCTURE format.

**Additional file 3. **Mantel correlograms for the relationship between genetic distance (*F*_*ST*_) and geographic distance (km) among *Penaeus monodon* populations (*n* = 7) using 10,624 SNPs.

**Additional file 4. **Genotypic data. Genotypes of 278 individuals of *P. monodon* at 89 adaptive genome-wide SNPs are included in a standard STRUCTURE format.

**Additional file 5. **Genotypic data. Genotypes of 278 individuals of *P. monodon* at 10,535 neutral genome-wide SNPs are included in a standard STRUCTURE format.

**Additional file 6. **Population differentiation estimates for *Penaeus monodon* populations sampled using neutral and outlier loci. Population pairwise *F*_*ST*_ (Weir & Cockerham, 1984) estimates computed by using the R package StAMPP v.1.5.1 (Pembleton et al., 2013). Pairwise *F*_*ST*_ values of 89 outlier loci are shown below the diagonal, and pairwise *F*_*ST*_ values of 10,535 neutral loci are reported above. All *F*_*ST*_ values were significant at *p* ≤ 0.05 following 1000 bootstraps performed across loci to generate confidence intervals. The only non-significant *F*_*ST*_ value (*p* > 0.05) is in bold type.

**Additional file 7. **Characterization of high-quality BLAST matches of outlier SNP to *Penaeus monodon* transcriptome contigs.

**Additional file 8.** Translation of eight outlier SNPs with transcriptome matches to protein sequences using all three reading frames.

**Additional file 9.** Sampling codes and site locations with number of individuals (N).

**Additional file 10. **Marine environmental variables (*n* = 23) for each of the seven geographically discrete sampling locations as determined using location-specific latitude and longitude.

## Data Availability

All data generated or analysed during this study are included in this published article and its Additional Files. The *P. monodon* transcriptome used for gene ontology in this study is available from the National Center for Biotechnology Information database under the following accession numbers (https://www.ncbi.nlm.nih.gov/nuccore/GGLH00000000.1): BioProject: PRJNA421400, BioSamples: SAMN08741487-SAMN08741521, SRA: SRP127068 (RR6868116-SRR6868172), TRA: GGLH00000000, (Huerlimann, et al. [72]).

## Abbreviations

AMOVA: Analysis of molecular variance
BIC: Bayesian information criterion
IBD: Isolation by distance
DAPC: Discriminant Analysis of Principal Components
SNP: Single nucleotide polymorphisms
PD: Population differentiation
EA: Environmental association
LD: Linkage disequilibrium
HWE: Hardy–Weinberg equilibrium
RDA: Redundancy analysis
pRDA: partial redundancy analysis
LFMM: Latent factor mixed models
FDR: False discovery rate
MRM: Multiple regression on distance matrices
PCA: Principal component analysis
*F*_*ST*_: Pairwise genetic differentiation values
CLCA: Calcium-activated chloride channel regulator
N_eLD_: Effective population size
kNN: Multiple K-nearest neighbor
