# Correction to: Fine-scale population structure and evidence for local adaptation in Australian giant black tiger shrimp (*Penaeus monodon*) using SNP analysis

**DOI:** 10.1186/s12864-021-07794-w

**Published:** 2021-07-20

**Authors:** Nga T. T. Vu, Kyall R. Zenger, Jarrod L. Guppy, Melony J. Sellars, Catarina N. S. Silva, Shannon R. Kjeldsen, Dean R. Jerry

**Affiliations:** 1 Australian Research Council Industrial Transformation Research Hub for Advanced Prawn Breeding, James Cook University, Townsville, QLD 4811 Australia; 2 Centre for Sustainable Tropical Fisheries and Aquaculture, College of Science and Engineering, James Cook University, Townsville, QLD 4811 Australia; 3 CSIRO Agriculture & Food, Integrated Sustainable Aquaculture Production Program, Queensland Bioscience Precinct, St Lucia, 4067 Australia; 4 Present address: Genics Pty Ltd, Level 5, Gehrmann Building. 60 Research Road, St Lucia, QLD 4067 Australia; 5 Tropical Futures Institute, James Cook University, Singapore, Singapore

**Correction to: BMC Genomics 21, 669 (2020)**

**https://doi.org/10.1186/s12864-020-07084-x**

After publication of this article [1], it is noticed the article contained an error: Fig. 3 erroneously contained panel B as a copy of panel A instead of the correct panel B illustration.

The correct Fig. 3 is presented in this Correction.

**Fig. 3:**
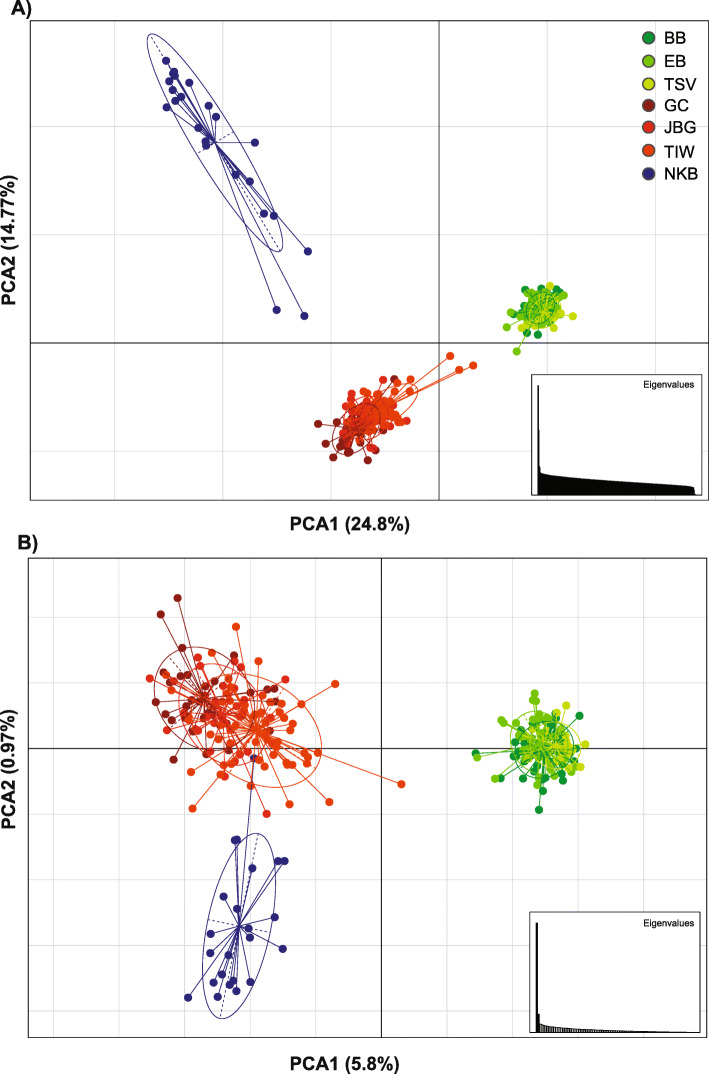
Principal components analysis based allele frequencies for (**a**) 10,535 neutral SNPs loci and (**b**) 89 outlier SNPs loci (BB: Bramston Beach, EB: Etty Bay, Townsville: TSV, Gulf of Carpentaria: GC, Joseph Bonaparte Gulf: JBG, Tiwi Island: TIW, and Nickol Bay: NKB)

The original article has been updated.
