# Supplementary material for: Fine-scale population structure and evidence for local adaptation in Australian giant black tiger shrimp (Penaeus monodon) using SNP analysis
Source: BMC Genomics. 2020 Sep 29;21:669. doi: 10.1186/s12864-020-07084-x (PMC7526253; doi:10.1186/s12864-020-07084-x)
Supplement: Supplementary file 1 — Additional file 1. SNP data analysis workflow from raw (n = 125,511) to final neutral (n = 10,535) and outlier (n = 89) loci datasets. [file 12864_2020_7084_MOESM1_ESM.pdf]

Additional file 1 Workflow of data analyses (from raw to final neutral and outlier loci datasets).

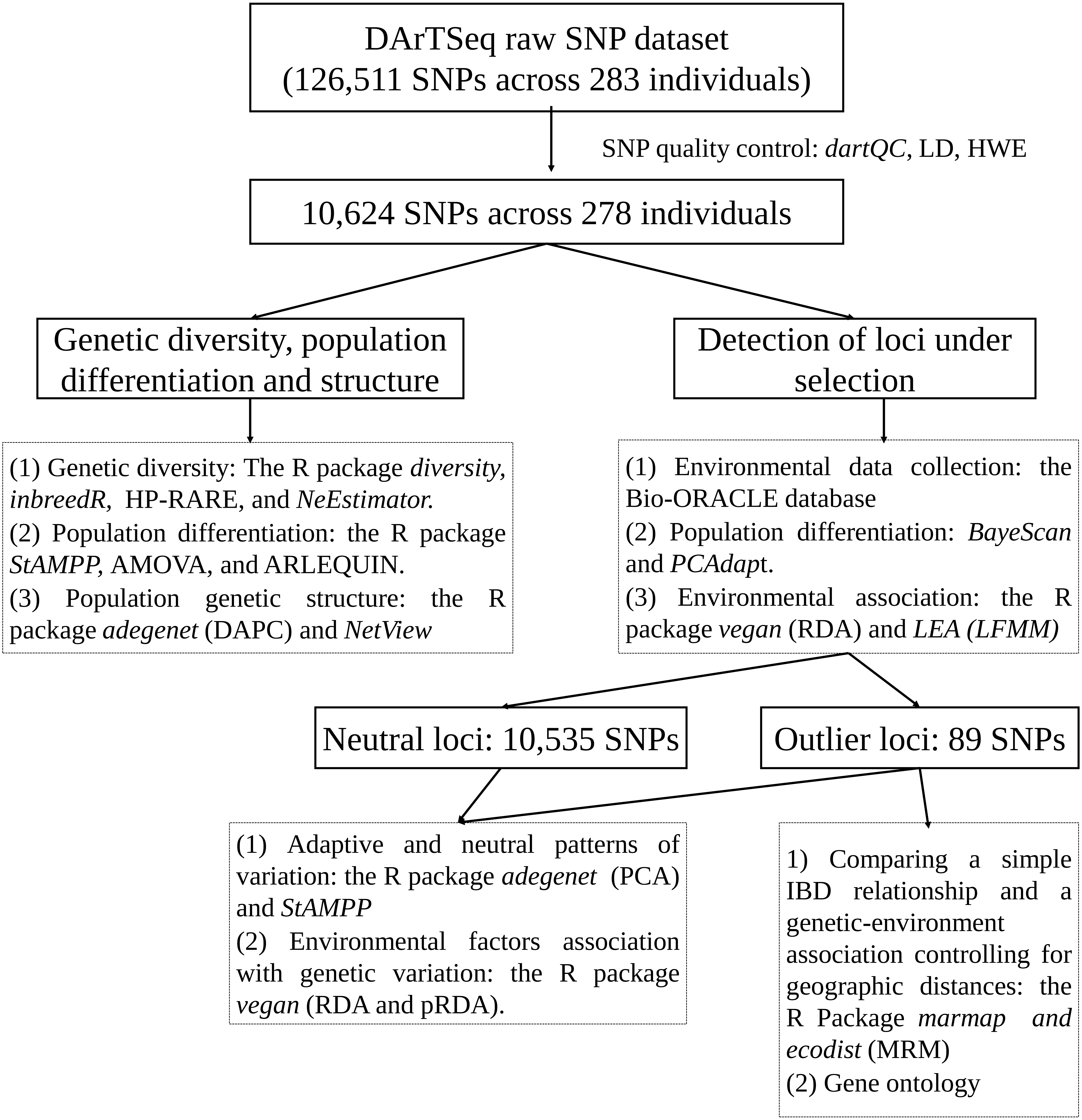

SNP quality control: (1) Average read depth of ≥ 7, (2) Average repeatability of ≥ 90%, (3) Call rate of ≥ 80%, (4) Similar sequence clusters of ≤ 0.95, (5) Minor allele frequency (MAF) of ≥ 0.02, (6) LD filters with a correlation coefficient (r^2) threshold of 0.2, (7) HWE filters ($p < 0.0001$), and (8) individuals with missing data (≤ 40%)
