# Supplementary material for: Fine-scale population structure and evidence for local adaptation in Australian giant black tiger shrimp (Penaeus monodon) using SNP analysis
Source: BMC Genomics. 2020 Sep 29;21:669. doi: 10.1186/s12864-020-07084-x (PMC7526253; doi:10.1186/s12864-020-07084-x)

Additional file 3 Mantel correlograms for the relationship between genetic distance (F_{ST}) and geographic distance (km) among *Penaeus monodon* populations (*n* = 7) using 10,624 SNPs.

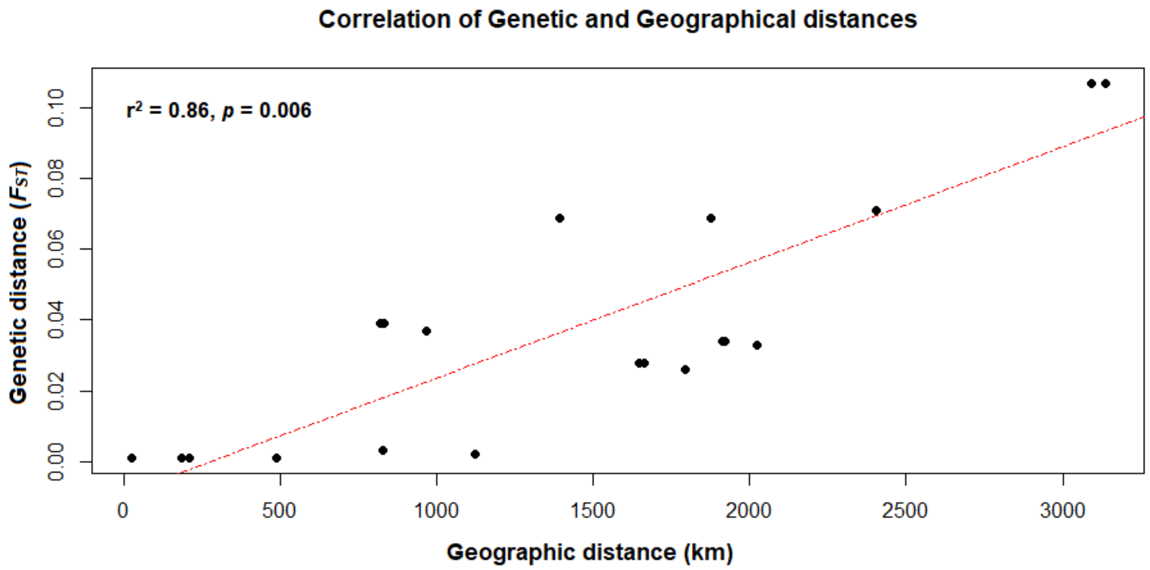

Supplement: Supplementary file 3 — Additional file 3. Mantel correlograms for the relationship between genetic distance (FST) and geographic distance (km) among Penaeus monodon populations (n = 7) using 10,624 SNPs. [file 12864_2020_7084_MOESM3_ESM.pdf]
