# Supplementary material for: Fine-scale population structure and evidence for local adaptation in Australian giant black tiger shrimp (Penaeus monodon) using SNP analysis
Source: BMC Genomics. 2020 Sep 29;21:669. doi: 10.1186/s12864-020-07084-x (PMC7526253; doi:10.1186/s12864-020-07084-x)
Supplement: Supplementary file 6 — Additional file 6. Population differentiation estimates for Penaeus monodon populations sampled using neutral and outlier loci. Population pairwise FST (Weir & Cockerham, 1984) estimates computed by using the R package StAMPP v.1.5.1 (Pembleton et al., 2013). Pairwise FST values of 89 outlier loci are shown below the diagonal, and pairwise FST values of 10,535 neutral loci are reported above. All FST values were significant at p ≤ 0.05 following 1000 bootstraps performed across loci to generate confidence intervals. The only non-significant FST value (p > 0.05) is in bold type. [file 12864_2020_7084_MOESM6_ESM.pdf]

Additional file 6 Population differentiation estimates for *Penaeus monodon* populations sampled using neutral and outlier loci

	**Bramston Beach**	**Etty Bay**	**Townsville**	**Gulf of Carpentaria**	**Joseph Bonaparte Gulf**	**Tiwi Island**	**Nickol Bay**
Bramston Beach	0	0.001	0.001	0.03	0.03	0.022	0.10
Etty Bay	**-0.001**	0	0.001	0.03	0.03	0.02	0.10
Townsville	**0.002**	**-0.005**	0	0.03	0.02	0.02	0.10
Gulf of Carpentaria	0.49	0.47	0.43	0	0.002	0.002	0.07
Joseph Bonaparte Gulf	0.46	0.44	0.40	0.01	0	0.001	0.07
Tiwi Island	0.38	0.36	0.33	0.03	0.02	0	0.07
Nickol Bay	0.64	0.63	0.60	0.22	0.21	0.19	0

Population pairwise F_{ST} (Weir & Cockerham, 1984) estimates computed by using the R package StAMPP v.1.5.1 (Pembleton *et al.*, 2013). Pairwise F_{ST} values of 89 outlier loci are shown below the diagonal, and pairwise F_{ST} values of 10,535 neutral loci are reported above. All F_{ST} values were significant at $p \leq 0.05$ following 1000 bootstraps performed across loci to generate confidence intervals. The only non-significant F_{ST} value ($p > 0.05$) is in bold type.
