# Supplementary material for: Fine-scale population structure and evidence for local adaptation in Australian giant black tiger shrimp (Penaeus monodon) using SNP analysis
Source: BMC Genomics. 2020 Sep 29;21:669. doi: 10.1186/s12864-020-07084-x (PMC7526253; doi:10.1186/s12864-020-07084-x)
Supplement: Supplementary file 7 — Additional file 7. Characterization of high-quality BLAST matches of outlier SNP to Penaeus monodon transcriptome contigs. [file 12864_2020_7084_MOESM7_ESM.pdf]

Additional file 7 Characterization of high-quality BLAST matchesof outlier SNP to *Penaeus monodon* transcriptome contigs [73].

SNP (#)	Contig matched	Pairwise Identity	Bit-Score	Contig Description	E Value	Hit end	Hit start	Query coverage (%)
PM1958_A	contig11442.1	100	128.5	calcium-activated chloride channel regulator 2-like	2.01E-29	2,039	1,971	100
	contig9994.1	100	128.5	Calcium-activated chloride channel regulator	2.01E-29	688	756	100
	contig3578.1	100	128.5	epithelial chloride channel -like	2.01E-29	3,761	3,693	100
	contig3237.1	100	128.5	epithelial chloride channel -like	2.01E-29	3,970	3,902	100
PM1958_B	contig11442.1	98.6	123.0	calcium-activated chloride channel regulator 2-like	9.33E-28	2,039	1,971	100
	contig9994.1	98.6	123.0	Calcium-activated chloride channel regulator	9.33E-28	688	756	100
	contig3578.1	98.6	123.0	epithelial chloride channel -like	9.33E-28	3,761	3,693	100
	contig3237.1	98.6	123.0	epithelial chloride channel -like	9.33E-28	3,970	3,902	100
PM6488_A	contig196702.1	97.1	58.4	---NA---	2.67E-08	283	316	49.28
	contig184051.1	97	56.5	---NA---	9.60E-08	160	192	47.83
	contig169503.1	100	62.1	---NA---	2.06E-09	293	261	47.83
	contig160248.1	100	62.1	---NA---	2.06E-09	112	144	47.83
	contig146699.1	97.1	58.4	---NA---	2.67E-08	259	292	49.28
	contig144611.1	97	56.5	---NA---	9.60E-08	402	370	47.83
	contig141018.1	97.1	58.4	---NA---	2.67E-08	118	85	49.28
	contig121911.1	97	56.5	---NA---	9.60E-08	160	192	47.83
	contig110726.1	97.1	58.4	---NA---	2.67E-08	7	40	49.28
	contig102320.1	97	56.5	---NA---	9.60E-08	203	235	47.83
	contig100878.1	97	56.5	---NA---	9.60E-08	451	483	47.83
	contig89079.1	100	60.2	---NA---	7.42E-09	510	479	46.38
	contig88989.1	97.1	58.4	---NA---	2.67E-08	152	119	49.28
	contig83954.1	97	56.5	---NA---	9.60E-08	479	511	47.83
	contig63011.1	97	56.5	---NA---	9.60E-08	535	503	47.83
	contig54766.1	100	60.2	---NA---	7.42E-09	813	782	46.38
	contig47238.2	97	56.5	---NA---	9.60E-08	419	451	47.83
	contig47238.1	97	56.5	---NA---	9.60E-08	419	451	47.83
	contig45025.1	100	54.7	---NA---	3.45E-07	484	456	42.03
	contig43670.1	100	60.2	---NA---	7.42E-09	495	464	46.38
	contig37913.1	100	60.2	---NA---	7.42E-09	183	152	46.38
	contig33852.1	100	60.2	---NA---	7.42E-09	783	752	46.38
	contig32241.1	100	54.7	---NA---	3.45E-07	484	456	42.03
	contig21545.1	97	56.5	---NA---	9.60E-08	1,731	1,763	47.83
	contig3381.1	97	56.5	AF445312_1unknown	9.60E-08	698	730	47.83
	contig1214.2	97	56.5	RNA-directed DNA polymerase from mobile element jockey-like	9.60E-08	159	191	47.83
	contig1214.1	97	56.5	RNA-directed DNA polymerase from mobile element jockey-like	9.60E-08	159	191	47.83
PM6488_B	contig221983.1	96.9	54.7	---NA---	3.45E-07	15	46	46.38
	contig215803.1	97	56.5	---NA---	9.60E-08	100	68	47.83
	contig211217.1	100	63.9	---NA---	5.74E-10	208	175	49.28
	contig209362.1	100	60.2	---NA---	7.42E-09	228	197	46.38
	contig196252.1	97	56.5	---NA---	9.60E-08	94	62	47.83
	contig191456.1	100	60.2	---NA---	7.42E-09	158	189	46.38
	contig190456.1	97.1	58.4	---NA---	2.67E-08	215	248	49.28
	contig183806.1	97	56.5	---NA---	9.60E-08	258	290	47.83
	contig182250.1	100	52.8	---NA---	1.24E-06	183	156	40.58
	contig174356.1	97	56.5	---NA---	9.60E-08	327	295	47.83
	contig173878.1	97	56.5	---NA---	9.60E-08	247	215	47.83
	contig170096.1	100	58.4	---NA---	2.67E-08	77	47	44.93
	contig168958.1	100	63.9	---NA---	5.74E-10	146	179	49.28
	contig165232.1	97	56.5	---NA---	9.60E-08	311	279	47.83
	contig160882.1	97.1	58.4	---NA---	2.67E-08	251	218	49.28
	contig160442.1	96.8	52.8	---NA---	1.24E-06	84	114	44.93
	contig155157.1	96.9	54.7	---NA---	3.45E-07	183	214	46.38
	contig153702.1	100	54.7	---NA---	3.45E-07	154	182	42.03
	contig151497.1	100	60.2	---NA---	7.42E-09	144	113	46.38
	contig150717.1	100	54.7	---NA---	3.45E-07	161	133	42.03
	contig146738.1	100	60.2	---NA---	7.42E-09	43	12	46.38
	contig145055.1	96.8	52.8	---NA---	1.24E-06	233	203	44.93
	contig144894.1	100	54.7	---NA---	3.45E-07	291	263	42.03
	contig144413.1	100	60.2	---NA---	7.42E-09	165	196	46.38
	contig142507.1	96.9	54.7	---NA---	3.45E-07	94	125	46.38
	contig141960.1	97	56.5	---NA---	9.60E-08	333	365	47.83
	contig141460.1	100	60.2	---NA---	7.42E-09	252	283	46.38
	contig137288.1	100	62.1	---NA---	2.06E-09	272	304	47.83
	contig136468.1	97	56.5	---NA---	9.60E-08	395	427	47.83
	contig134383.1	97	56.5	---NA---	9.60E-08	306	274	47.83
	contig133091.1	96.8	52.8	---NA---	1.24E-06	163	193	44.93
	contig131883.1	100	56.5	---NA---	9.60E-08	278	249	43.48
	contig131661.1	97	56.5	---NA---	9.60E-08	370	338	47.83
	contig130490.1	100	58.4	---NA---	2.67E-08	356	386	44.93
	contig129639.1	100	58.4	---NA---	2.67E-08	373	343	44.93
	contig127989.1	100	54.7	---NA---	3.45E-07	218	190	42.03
	contig126961.1	100	63.9	---NA---	5.74E-10	153	186	49.28
	contig123773.1	100	60.2	---NA---	7.42E-09	222	253	46.38
	contig120030.1	97	56.5	---NA---	9.60E-08	335	303	47.83
	contig119207.1	100	60.2	---NA---	7.42E-09	285	316	46.38
	contig114246.1	100	62.1	---NA---	2.06E-09	233	265	47.83
	contig110605.1	100	60.2	---NA---	7.42E-09	152	121	46.38
	contig110569.1	100	62.1	---NA---	2.06E-09	368	400	47.83
	contig108609.1	97	56.5	---NA---	9.60E-08	459	427	47.83
	contig108525.2	100	60.2	---NA---	7.42E-09	266	297	46.38
	contig108525.1	100	60.2	---NA---	7.42E-09	267	298	46.38
	contig108273.1	97	56.5	---NA---	9.60E-08	60	28	47.83
	contig106547.1	97	56.5	---NA---	9.60E-08	285	317	47.83
	contig106521.1	100	54.7	---NA---	3.45E-07	239	211	42.03
	contig102310.1	100	60.2	---NA---	7.42E-09	526	495	46.38
	contig102032.1	96.9	54.7	---NA---	3.45E-07	159	128	46.38
	contig98550.1	100	56.5	---NA---	9.60E-08	324	295	43.48
	contig95902.1	97	56.5	---NA---	9.60E-08	117	85	47.83
	contig91117.1	100	60.2	---NA---	7.42E-09	307	276	46.38
	contig90378.1	100	54.7	---NA---	3.45E-07	46	74	42.03
	contig88653.1	100	60.2	---NA---	7.42E-09	489	520	46.38
	contig88268.1	100	60.2	---NA---	7.42E-09	64	33	46.38
	contig85300.1	96.9	54.7	---NA---	3.45E-07	331	300	46.38
	contig85217.1	100	58.4	---NA---	2.67E-08	378	408	44.93
	contig84904.1	100	60.2	---NA---	7.42E-09	335	304	46.38
	contig84045.1	100	60.2	---NA---	7.42E-09	275	306	46.38
	contig83472.1	97	56.5	---NA---	9.60E-08	211	179	47.83
	contig79424.1	96.9	54.7	---NA---	3.45E-07	392	423	46.38
	contig79102.1	100	60.2	---NA---	7.42E-09	282	313	46.38
	contig75907.1	100	56.5	---NA---	9.60E-08	657	686	43.48
	contig75105.1	100	52.8	---NA---	1.24E-06	698	725	40.58
	contig75049.1	97	56.5	---NA---	9.60E-08	131	99	47.83
	contig74027.1	97.1	58.4	---NA---	2.67E-08	433	466	49.28
	contig70385.1	96.9	54.7	---NA---	3.45E-07	537	506	46.38
	contig69377.1	97	56.5	---NA---	9.60E-08	693	725	47.83
	contig69263.1	100	54.7	---NA---	3.45E-07	491	463	42.03
	contig67491.1	96.9	54.7	---NA---	3.45E-07	51	82	46.38
	contig65678.1	100	54.7	---NA---	3.45E-07	665	637	42.03
	contig63233.1	100	62.1	---NA---	2.06E-09	744	712	47.83
	contig60751.1	100	60.2	---NA---	7.42E-09	167	136	46.38
	contig59941.1	100	60.2	---NA---	7.42E-09	84	53	46.38
	contig58971.1	97.1	58.4	---NA---	2.67E-08	124	91	49.28
	contig56349.1	100	60.2	---NA---	7.42E-09	765	734	46.38
	contig55153.1	97	56.5	---NA---	9.60E-08	263	231	47.83
	contig54969.1	97	56.5	---NA---	9.60E-08	726	758	47.83
	contig54325.1	100	54.7	---NA---	3.45E-07	674	646	42.03
	contig51877.1	100	52.8	---NA---	1.24E-06	396	369	40.58
	contig49724.1	100	60.2	---NA---	7.42E-09	468	437	46.38
	contig47238.2	97	56.5	---NA---	9.60E-08	713	745	47.83
	contig47238.1	97	56.5	---NA---	9.60E-08	852	884	47.83
	contig39463.2	100	54.7	---NA---	3.45E-07	747	719	42.03
	contig34890.1	100	54.7	---NA---	3.45E-07	161	133	42.03
	contig33357.1	100	62.1	---NA---	2.06E-09	1,360	1,392	47.83
	contig29020.1	100	54.7	---NA---	3.45E-07	1,314	1,342	42.03
	contig23832.1	96.8	52.8	---NA---	1.24E-06	1,479	1,509	44.93
	contig21081.1	96.9	54.7	---NA---	3.45E-07	837	806	46.38
	contig19487.1	100	56.5	---NA---	9.60E-08	1,328	1,299	43.48
	contig15653.1	100	56.5	---NA---	9.60E-08	88	59	43.48
	contig14585.1	100	54.7	---NA---	3.45E-07	1,202	1,174	42.03
	contig13069.1	100	54.7	---NA---	3.45E-07	684	656	42.03
	contig11529.1	100	60.2	---NA---	7.42E-09	428	459	46.38
	contig9666.1	96.9	54.7	calcium-activated potassium channel slowpoke isoform X7	3.45E-07	3	34	46.38
	contig8956.1	100	54.7	---NA---	3.45E-07	370	342	42.03
PM1771_A	contig169701.1	95.9	78.7	RNA-directed DNA polymerase from mobile element jockey	2.05E-14	69	21	69.57
	contig162921.1	94.1	76.8	---NA---	7.37E-14	51	1	72.46
	contig155224.1	97.1	58.4	---NA---	2.67E-08	103	69	49.28
	contig123561.1	100	54.7	---NA---	3.45E-07	32	4	42.03
	contig72709.1	96.1	82.4	RNA-directed DNA polymerase from mobile element jockey	1.58E-15	249	299	72.46
	contig41955.1	96.1	82.4	RNA-directed DNA polymerase from mobile element jockey-like	1.58E-15	463	513	72.46
	contig32221.1	87.8	80.5	RNA-directed DNA polymerase from mobile element jockey	5.70E-15	1,490	1,417	95.65
	contig29186.1	96.1	82.4	RNA-directed DNA polymerase from mobile element jockey-like	1.58E-15	1,066	1,116	72.46
	contig22935.1	100	54.7	rna-directed dna polymerase from mobile element	3.45E-07	906	878	42.03
	contig17548.1	96.1	82.4	RNA-directed DNA polymerase from mobile element jockey-like	1.58E-15	1,314	1,364	72.46
	contig8125.3	94.1	76.8	RNA-directed DNA polymerase from mobile element jockey-like	7.37E-14	355	405	72.46
	contig8125.2	96.1	82.4	RNA-directed DNA polymerase from mobile element jockey	1.58E-15	1,722	1,772	72.46
	contig8125.1	96.1	82.4	rna-directed dna polymerase from mobile element jockey	1.58E-15	2,316	2,366	72.46
	contig8082.1	94.1	76.8	lian-aal retrotransposon	7.37E-14	2,428	2,478	72.46
	contig4273.1	87.1	73.1	RNA-directed DNA polymerase from mobile element jockey-like	9.53E-13	2,488	2,419	89.86
	contig3120.1	96.1	82.4	rna-directed dna polymerase from mobile element jockey	1.58E-15	1,491	1,441	72.46
PM1771_B	contig169701.1	95.9	78.7	RNA-directed DNA polymerase from mobile element jockey	2.05E-14	69	21	69.57
	contig162921.1	94.1	76.8	---NA---	7.37E-14	51	1	72.46
	contig155224.1	97.1	58.4	---NA---	2.67E-08	103	69	49.28
	contig123561.1	100	54.7	---NA---	3.45E-07	32	4	42.03
	contig72709.1	96.1	82.4	RNA-directed DNA polymerase from mobile element jockey	1.58E-15	249	299	72.46
	contig41955.1	96.1	82.4	RNA-directed DNA polymerase from mobile element jockey-like	1.58E-15	463	513	72.46
	contig32221.1	95.9	78.7	RNA-directed DNA polymerase from mobile element jockey	2.05E-14	1,490	1,442	69.57
	contig29186.1	96.1	82.4	RNA-directed DNA polymerase from mobile element jockey-like	1.58E-15	1,066	1,116	72.46
	contig22935.1	100	54.7	rna-directed dna polymerase from mobile element	3.45E-07	906	878	42.03
	contig17548.1	96.1	82.4	RNA-directed DNA polymerase from mobile element jockey-like	1.58E-15	1,314	1,364	72.46
	contig8125.3	94.1	76.8	RNA-directed DNA polymerase from mobile element jockey-like	7.37E-14	355	405	72.46
	contig8125.2	96.1	82.4	RNA-directed DNA polymerase from mobile element jockey	1.58E-15	1,722	1,772	72.46
	contig8125.1	96.1	82.4	rna-directed dna polymerase from mobile element jockey	1.58E-15	2,316	2,366	72.46
	contig8082.1	86.8	78.7	lian-aal retrotransposon	2.05E-14	2,428	2,503	98.55
	contig4273.1	95.6	71.3	RNA-directed DNA polymerase from mobile element jockey-like	3.43E-12	2,488	2,444	63.77
	contig3120.1	96.1	82.4	rna-directed dna polymerase from mobile element jockey	1.58E-15	1,491	1,441	72.46
PM4714_A	contig5062.3	100	121.2	PREDICTED: uncharacterized protein LOC108744461 isoform X	3.36E-27	348	412	94.2
	contig5062.2	100	121.2	Uncharacterized protein APZ42_034504	3.36E-27	335	399	94.2
	contig5062.1	100	121.2	Uncharacterized protein APZ42_034504	3.36E-27	1,169	1,233	94.2
PM4714_A	contig5062.3	98.5	115.6	PREDICTED: uncharacterized protein LOC108744461 isoform X	1.56E-25	348	412	94.2
	contig5062.2	98.5	115.6	Uncharacterized protein APZ42_034504	1.56E-25	335	399	94.2
	contig5062.1	98.5	115.6	Uncharacterized protein APZ42_034504	1.56E-25	1,169	1,233	94.2
PM3856_A	contig9977.1	100	54.7	kinase pkn|GO:0008484 GO:0008152	3.45E-07	857	885	42.03
	contig2078.1	100	54.7	kinase pkn|GO:0008484 GO:0008152	3.45E-07	1,459	1,487	42.03
PM3856_B	contig14798.1	100	54.7	kinase pkn|GO:0008484 GO:0008152	3.45E-07	2,223	2,195	42.03
**PM10591_A**	NO HIT							0
**PM10591_B**	contig113267.1	100	62.1	---NA---	2.06E-09	156	188	47.83
PM6057_A	contig170249.1	91.5	82.4	endonuclease-reverse transcriptase|GO:0003964 GO:0006278	1.58E-15	124	66	85.51
PM6057_B	contig212013.1	91.4	80.5	endonuclease-reverse transcriptase|GO:0003964 GO:0006278	5.70E-15	59	2	84.06
	contig210575.1	88.1	71.3	endonuclease-reverse transcriptase|GO:0003964 GO:0006278	3.43E-12	87	29	85.51
	contig195976.1	89.8	76.8	endonuclease-reverse transcriptase|GO:0003964 GO:0006278	7.37E-14	78	20	85.51
	contig191278.1	94.9	62.1	endonuclease-reverse transcriptase|GO:0003964 GO:0006278	2.06E-09	338	300	56.52
	contig151251.1	91.1	76.8	RNA-directed DNA polymerase from mobile element jockey| GO:0003964 GO:0006278	7.37E-14	389	334	81.16
	contig108767.1	89.8	76.8	endonuclease-reverse transcriptase|GO:0003964 GO:0006278	7.37E-14	177	119	85.51
	contig100612.1	91.4	80.5	endonuclease-reverse transcriptase|GO:0003964 GO:0006278	5.70E-15	354	297	84.06
	contig99594.1	91.1	76.8	endonuclease-reverse transcriptase|GO:0003964 GO:0006278	7.37E-14	352	407	81.16
	contig20642.1	89.7	75.0	endonuclease-reverse transcriptase	2.65E-13	766	709	84.06
	contig15540.1	92.9	82.4	endonuclease-reverse transcriptase	1.58E-15	1,723	1,778	81.16
	contig2742.1	90.9	58.4	endonuclease-reverse transcriptase|GO:0003964 GO:0006278	2.67E-08	2,057	2,015	63.77
**PM2065_A**	contig160882.1	93.5	67.6	---NA---	4.44E-11	156	112	66.67
**PM2065_B**	NO HIT							0
PM5846_A	contig69233.1	91.9	87.9	---NA---	3.40E-17	774	713	89.86
PM5846_B	contig69233.1	90.3	82.4	---NA---	1.58E-15	774	713	89.86
**PM10621_A**	NO HIT							0
**PM10621_B**	contig83159.1	95.2	67.6	RNA-directed DNA polymerase from mobile element| GO:0003824 GO:0005488 GO:0044260 GO:0090304	4.44E-11	1	42	60.87
	contig53029.1	95.9	80.5	RNA-directed DNA polymerase from mobile element	5.70E-15	1,011	963	71.01
	contig27951.1	93.9	75.0	RNA-directed DNA polymerase from mobile element jockey-like| GO:0003824 GO:0005488 GO:0044260 GO:0090304	2.65E-13	558	510	71.01
	contig17322.1	93.9	75.0	RNA-directed DNA polymerase from mobile element	2.65E-13	235	283	71.01
PM1296_A	contig46386.1	97.3	63.9	venom protease-like|GO:0008233	5.74E-10	287	251	53.62
	contig40687.1	97.3	63.9	venom protease-like|GO:0008233	5.74E-10	270	234	53.62
	contig21273.1	97.3	63.9	venom protease-like|GO:0008233	5.74E-10	275	239	53.62
	contig17797.1	97.3	63.9	venom protease-like|GO:0008233	5.74E-10	326	290	53.62
	contig6796.1	97.3	63.9	prophenoloxidase activating factor 1| GO:0004252 GO:0006508	5.74E-10	3,195	3,231	53.62
PM1296_B	contig38125.1	97.3	63.9	venom protease-like|GO:0008233	5.74E-10	359	323	53.62

Two alleles generated by outlier SNP denoted as "_A" and "_B"; Shading denotes outlier SNP with only one allele that matched *P. monodon* transcriptome.
