# Supplementary material for: Fine-scale population structure and evidence for local adaptation in Australian giant black tiger shrimp (Penaeus monodon) using SNP analysis
Source: BMC Genomics. 2020 Sep 29;21:669. doi: 10.1186/s12864-020-07084-x (PMC7526253; doi:10.1186/s12864-020-07084-x)
Supplement: Supplementary file 8 — Additional file 8. Translation of eight outlier SNPs with transcriptome matches to protein sequences using all three reading frames. [file 12864_2020_7084_MOESM8_ESM.pdf]

Additional file 8 Translation of eight outlier SNP with transcriptome matches to protein sequences using all three reading frames.

SNP ID	Sequence (5' - 3')	Translation Frame 1 (5' - 3')	Translation Frame 2 (5' - 3')	Translation Frame 3 (5' - 3')	Translation Frame 1 (3' - 5')	Translation Frame 2 (3' - 5')	Translation Frame 3 (3' - 5')
PM1958_A	TGCAGACGCGTACGCCGCCTACCCGTACCCCTACCACCCAGGCTACAGCCACGAGGAGCTGCCCCCATA	CRRVRRLPVPLPPRLQPRGAAPI	**ADAYAAYPYPYHPGYSHEELPP**	QTRTPPTRTPTTQATATRSCPH	**YGGSSSWL*PGW*GYG*AAYASA**	MGAAPRGCSLGGRGTGRRRTRL	WGQLLVAVAWVVGVRVGGVRVC
PM1958_B	TGCAGACGCGTACGCCGCCTACCCATACCCCTACCACCCAGGCTACAGCCACGAGGAGCTGCCCCCATA	CRRVRRLPIPLPPRLQPRGAAPI	**ADAYAAYPYPYHPGYSHEELPP**	QTRTPPTHTPTTQATATRSCPH	**YGGSSSWL*PGW*GYG*AAYASA**	MGAAPRGCSLGGRGMGRRRTRL	WGQLLVAVAWVVGVWVGGVRVC
PM6488_A	TGCAGGTACCAGTCACCCCAGGATGCTGTGCATGATCCGAGATCGGAAGAGCGGTTCAGCAGGAATGCC	**CRYQSPQDAVHDPRSEERFSRNA**	AGTSHPRMLCMIRDRKSGSAGM	QVPVTPGCCA*SEIGRAVQQEC	GIPAEPLFRSRIMHSILG*LVPA	**AFLLNRSSDLGSCTASWGDWYL**	HSC*TALPISDHAQHPGVTGTC
PM6488_B	TGCAGGTACCAGTCGCCCCAGGATGCTGTGCATGATCCGAGATCGGAAGAGCGGTTCAGCAGGAATGCC	**CRYQSPQDAVHDPRSEERFSRNA**	AGTSRPRMLCMIRDRKSGSAGM	QVPVAPGCCA*SEIGRAVQQEC	GIPAEPLFRSRIMHSILGRLVPA	**AFLLNRSSDLGSCTASWGDWYL**	HSC*TALPISDHAQHPGATGTC
PM1771_A	TGCAGCACACGACCGAGTGGGGGGACTTCAATGCACACCACCCCATCCTGGCTCCCTACAGGGCACCGA	CSTRPSGGTSMHTTPSWLPTGHR	AAHDRVGGLQCTPPHPGSLQGT	**QHTTEWGDFNAHHPILAPYRAP**	SVPCREPGWGGVH*SPPTRSCAA	RCPVGSQDGVVCIEVPPLGRVL	**GAL*GARMGWCALKSPHSVVCC**
PM1771_B	TGCAGCACACGACTGAGTGGGGGGACTTCAATGCACACCACCCCATCCTGGCTCCCTACAGGGCACCGA	CSTRLSGGTSMHTTPSWLPTGHR	AAHD*VGGLQCTPPHPGSLQGT	**QHTTEWGDFNAHHPILAPYRAP**	SVPCREPGWGGVH*SPPTQSCAA	RCPVGSQDGVVCIEVPPLSRVL	**GAL*GARMGWCALKSPHSVVCC**
PM4714_A	TGCAGTACATAAGGCACAGCGAGCTGGACGGGCACTCGCGGCCGCACTTGTAGCACACGAAGCCGAGAT	CST*GTASWTGTRGRTCSTRSRD	AVHKAQRAGRALAAALVAHEAE	**QYIRHSELDGHSRPHL*HTKPR**	ISASCATSAAASARPARCALCTA	SRLRVLQVRPRVPVQLAVPYVL	**LGFVCYKCGRECPSSSLCLMYC**
PM4714_B	TGCAGTACATCAGGCACAGCGAGCTGGACGGGCACTCGCGGCCGCACTTGTAGCACACGAAGCCGAGAT	CSTSGTASWTGTRGRTCSTRSRD	AVHQAQRAGRALAAALVAHEAE	**QYIRHSELDGHSRPHL*HTKPR**	ISASCATSAAASARPARCA*CTA	SRLRVLQVRPRVPVQLAVPDVL	**LGFVCYKCGRECPSSSLCLMYC**
PM3856_A	TGCAGCTGGGAATCCTGCTGTGTCTGCCGAGATCGGAAGAGCGGTTCAGCAGGAATGCCGAGACCGATC	CSWESCCVCRDRKSGSAGMPRPI	AAGNPAVSAEIGRAVQQECRDR	QLGILLCLPRSEERFSRNAETD	DRSRHSC*TALPISADTAGFPAA	IGLGIPAEPLFRSRQTQQDSQL	SVSAFLLNRSSDLGRHSRIPSC
PM3856_B	TGCAGCTGTGAATCCTGCTGTGTCTGCCGAGATCGGAAGAGCGGTTCAGCAGGAATGCCGAGACCGATC	CSCESCCVCRDRKSGSAGMPRPI	AAVNPAVSAEIGRAVQQECRDR	QL*ILLCLPRSEERFSRNAETD	DRSRHSC*TALPISADTAGFTAA	IGLGIPAEPLFRSRQTQQDSQL	SVSAFLLNRSSDLGRHSRIHSC
PM6057_A	TGCAGCAACTACTGTGACATTACACTGCTCAGTATACCAGACAAGATTTTCACCCACATCCGTGATAAC	CSNYCDITLLSIPDKIFTHIRDN	AATTVTLHCSVYQTRFSPTSVI	QQLL*HYTAQYTRQDFHPHP**	VITDVGENLVWYTEQCNVTVVAA	LSRMWVKILSGILSSVMSQ*LL	**YHGCG*KSCLVY*AV*CHSSCC**
PM6057_B	TGCAGCAACTACTGTGGCATTACACTGCTCAGTATACCAGACAAGATTTTCACCCACATCCGTGATAAC	CSNYCGITLLSIPDKIFTHIRDN	AATTVALHCSVYQTRFSPTSVI	QQLLWHYTAQYTRQDFHPHP**	VITDVGENLVWYTEQCNATVVAA	LSRMWVKILSGILSSVMPQ*LL	**YHGCG*KSCLVY*AV*CHSSCC**
PM5846_A	TGCAGCTTCAGTCCAGGCCACAAATTTCTGTTGCTTTGTCCAATGAGATTAATTACAGCGTTCGGCATG	CSFSPGHKFLLLCPMRLITAFGM	AASVQATNFCCFVQ*D*LQRSA	QLQSRPQISVALSNEINYSVRH	HAERCN*SHWTKQQKFVAWTEAA	**MPNAVINLIGQSNRNLWPGLKL**	CRTL*LISLDKATEICGLD*SC
PM5846_B	TGCAGATTCAGTCCAGGCCACAAATTTCTGTTGCTTTGTCCAATGAGATTAATTACAGCGTTCGGCATG	CRFSPGHKFLLLCPMRLITAFGM	ADSVQATNFCCFVQ*D*LQRSA	QIQSRPQISVALSNEINYSVRH	HAERCN*SHWTKQQKFVAWTESA	**MPNAVINLIGQSNRNLWPGLNL**	CRTL*LISLDKATEICGLD*IC
PM10621_A	TGCAGGATATCAAGCAAGGGACGGCGCCCCCGAGATCGGAAGAGCGGTTCAGCAGGAATGCCGAGACCG	CRISSKGRRPRDRKSGSAGMPRP	**AGYQARDGAPEIGRAVQQECRD**	QDIKQGTAPPRSEERFSRNAET	**RSRHSC*TALPISGAPSLA*YPA**	GLGIPAEPLFRSRGRRPLLDIL	VSAFLLNRSSDLGGAVPCLISC
PM10621_B	TGCAGGATATCAAGCAAGGGACGGCGCTCCCGAGATCGGAAGAGCGGTTCAGCAGGAATGCCGAGACCG	CRISSKGRRSRDRKSGSAGMPRP	**AGYQARDGAPEIGRAVQQECRD**	QDIKQGTALPRSEERFSRNAET	**RSRHSC*TALPISGAPSLA*YPA**	GLGIPAEPLFRSRERRPLLDIL	VSAFLLNRSSDLGSAVPCLISC

Two alleles generated by each outlier SNP denoted as "_A" and "_B"; Red characters indicate SNP locations within each nucleotide or translated protein sequence; Protein translation frames in which SNP caused synonymous mutations are bolded; Asterisks (*) denote stop codons.
