# Supplementary material for: Fine-scale population structure and evidence for local adaptation in Australian giant black tiger shrimp (Penaeus monodon) using SNP analysis
Source: BMC Genomics. 2020 Sep 29;21:669. doi: 10.1186/s12864-020-07084-x (PMC7526253; doi:10.1186/s12864-020-07084-x)
Supplement: Supplementary file 9 — Additional file 9. Sampling codes and site locations with number of individuals (N). [file 12864_2020_7084_MOESM9_ESM.pdf]

Additional file 9 Sampling codes and site locations with number of individuals (N).

Code	Sampling locations	Regions	N	Latitude	Longitude
BB	Bramston Beach	north Queensland	60	-17.353	146.0527
EB	Etty Bay	north Queensland	50	-17.5617	146.109
TSV	Townsville	north Queensland	22	-19.0821	146.8175
GC	Gulf of Carpentaria	Northern Territory	35	-14.9977	138.7808
JBG	Joseph Bonaparte Gulf	Northern Territory	34	-14.6818	128.3714
TIW	Tiwi Island	Northern Territory	56	-11.8668	131.8315
NKB	Nickol Bay	Western Australia	26	-20.697	116.8632
