# Supplementary material for: Fine-scale population structure and evidence for local adaptation in Australian giant black tiger shrimp (Penaeus monodon) using SNP analysis
Source: BMC Genomics. 2020 Sep 29;21:669. doi: 10.1186/s12864-020-07084-x (PMC7526253; doi:10.1186/s12864-020-07084-x)
Supplement: Supplementary file 10 — Additional file 10. Marine environmental variables (n = 23) for each of the seven geographically discrete sampling locations as determined using location-specific latitude and longitude. [file 12864_2020_7084_MOESM10_ESM.pdf]

Additional file 10 Marine environmental variables (n = 23) for each of the seven geographically discrete sampling locations as determined using location-specific latitude and longitude.

State	Northern Territory	Northern Territory	Northern Territory	North Queensland	North Queensland	North Queensland	Western Australia
Sampling Region	Gulf of Carpentaria	Joseph Bonaparte Gulf	Tiwi Island	Etty Bay	Bramston Beach	Townsville	Nickol Bay
Code	GC	JBG	TIW	EB	BB	TSV	NKB
Time period and grain	2000 - 2014	2000 - 2014	2000 - 2014	2000 - 2014	2000 - 2014	2000 - 2014	2000 - 2014
Latitude	-14.997655	-14.681809	-11.866834	-17.561792	-17.353008	-19.082107	-20.697014
Longitude	138.780787	128.371358	131.83147	146.108953	146.052726	146.817537	116.863229
Surface Salinity Min (Env_1)	33.05	30.01	29.96	34.05	34.05	33.73	32.94
Surface Temperature Max (Env_2)	30.94	31.29	30.92	29.54	29.54	29.32	31.48
Surface Temperature Min (Env_3)	23.44	21.82	24.02	22.54	22.54	21.92	21.25
Surface Current velocity Mean (Env_4)	0.14	0.03	0.05	0.05	0.05	0.16	0.31
Surface Phytoplankton Mean (Env_5)	1.28	1.45	1.52	1.44	1.44	1.67	1.39
Benthic Temperature Mean (Env_6)	0.14	0.02	0.02	0.05	0.05	0.04	0.05
Benthic Current velocity Mean (Env_7)	0.03	0.03	0.03	0.03	0.03	0.02	0.05
Surface Salinity Max	35.89	35.55	35.54	35.50	35.50	35.80	35.62
Surface Salinity Mean	34.66	33.79	33.13	35.05	35.05	35.19	34.55
Surface Dissolved molecular oxygen Max	227.83	220.27	217.21	227.29	227.29	236.35	227.83
Surface Dissolved molecular oxygen Min	187.51	189.57	190.44	193.65	193.65	196.70	193.00
Surface Current velocity Max	0.06	0.18	0.22	0.40	0.40	0.22	0.15
Surface Current velocity Min	0.04	0.16	0.17	0.37	0.37	0.35	0.04
Surface Phytoplankton Min	1.01	1.10	1.24	1.44	1.44	1.20	1.11
Benthic Salinity Max	35.78	36.09	34.76	35.36	35.36	35.44	35.27
Benthic Salinity Mean	34.41	35.34	34.39	35.02	35.02	35.08	34.92
Benthic Salinity Min	33.03	34.54	33.79	34.48	34.48	34.50	34.42
Benthic Temperature Max	0.51	0.11	0.13	0.33	0.33	0.22	0.13
Benthic Dissolved molecular oxygen Max	211.28	219.13	218.71	219.39	219.39	233.37	224.18
Benthic Dissolved molecular oxygen Mean	201.67	201.81	201.89	207.09	207.09	210.38	207.47
Benthic Current velocity Max	0.21	0.11	0.14	0.32	0.32	0.23	0.13
Benthic Phytoplankton Mean	0.98	1.12	1.13	1.21	1.21	1.28	1.31
Benthic Phytoplankton Min	0.72	0.90	0.91	0.94	0.94	0.97	1.04
